# Strengthening grapevine berry physiology and composition through kaolin–silicon foliar applications under summer stress in mediterranean vineyard

**DOI:** 10.3389/fpls.2026.1906889

**Published:** 2026-08-12

**Authors:** Ana Monteiro, Sandra Pereira, Helena Ferreira, Rebeca Cruz, Susana Casal, A. Sergio Serrano, Ricardo Silva, Emanuel Lopes, Joana Valente, Fernando Alves, José Moutinho-Pereira, Lia-Tânia Dinis

**Affiliations:** 1 CITAB - Centre for the Research and Technology of Agro-Environmental and Biological Sciences, University of Trás-os-Montes and Alto Douro (UTAD), Vila Real, Portugal; 2 Inov4Agro - Institute for Innovation, Capacity Building and Sustainability of Agri-Food Production, University of Trás-os-Montes and Alto Douro (UTAD), Vila Real, Portugal; 3 LAQV-REQUIMTE, Department of Chemical Sciences, Faculty of Pharmacy, University of Porto, Porto, Portugal; 4 Regional Institute for Agri-Food and Forestry. Research and Development of Castilla-La Mancha (IRIAF), Tomelloso, Spain; 5 Symington Family Estates, Vila Nova de Gaia, Portugal

**Keywords:** abiotic stress mitigation, berry quality, berry waxes, climate change, cuticular triterpenoids, Mediterranean viticulture, Touriga Nacional

## Abstract

**Introduction:**

The increasing frequency of heat and drought events in Mediterranean vineyards threatens vine performance and berry quality. Kaolin (Kl) particle films combined with silicon have been proposed as mitigation strategies to reduce temperature and improve plant water status; however, their effects on berry structure and metabolism remain uncertain.

**Methods:**

This study evaluated the foliar application of MiKS, a formulation combining 2% kaolin (Kl) with increasing silicon concentrations (2–8%, v/v), on berry skin structure, cuticular wax composition, metabolite profile, and wine volatile composition of the Touriga Nacional variety grown in the Douro region over two growing seasons.

**Results and discussion:**

MiKS treatments promoted significant structural reinforcement of berry skin by increasing cuticle thickness at both veraison and harvest. These anatomical changes were associated with a substantial remodeling of cuticular wax composition, characterized by a predominance of pentacyclic triterpenoids, particularly oleanolic acid, and a reduction in the relative abundance of carboxylic acids. Berry metabolism responded in a stage- and season-dependent manner, with changes in phenolic compounds, organic acids, amino acid-related metabolites, and flavan-3-ols. Carbon isotope discrimination (δ¹³C) indicated improved water status under MiKS 2, whereas technological ripening remained balanced. MiKS treatments also reduced volatile acidity and altered wine volatile organic compound profiles, with higher silicon concentrations promoting esters and alcohols associated with fruity and floral aroma characteristics.

**Conclusion:**

The combined application of kaolin and silicon promoted coordinated structural, physiological, metabolic, and oenological responses while preserving grape technological quality, highlighting MiKS as a promising sustainable foliar strategy to improve grapevine resilience under Mediterranean summer stress.

## Graphical abstract

**Figure:**
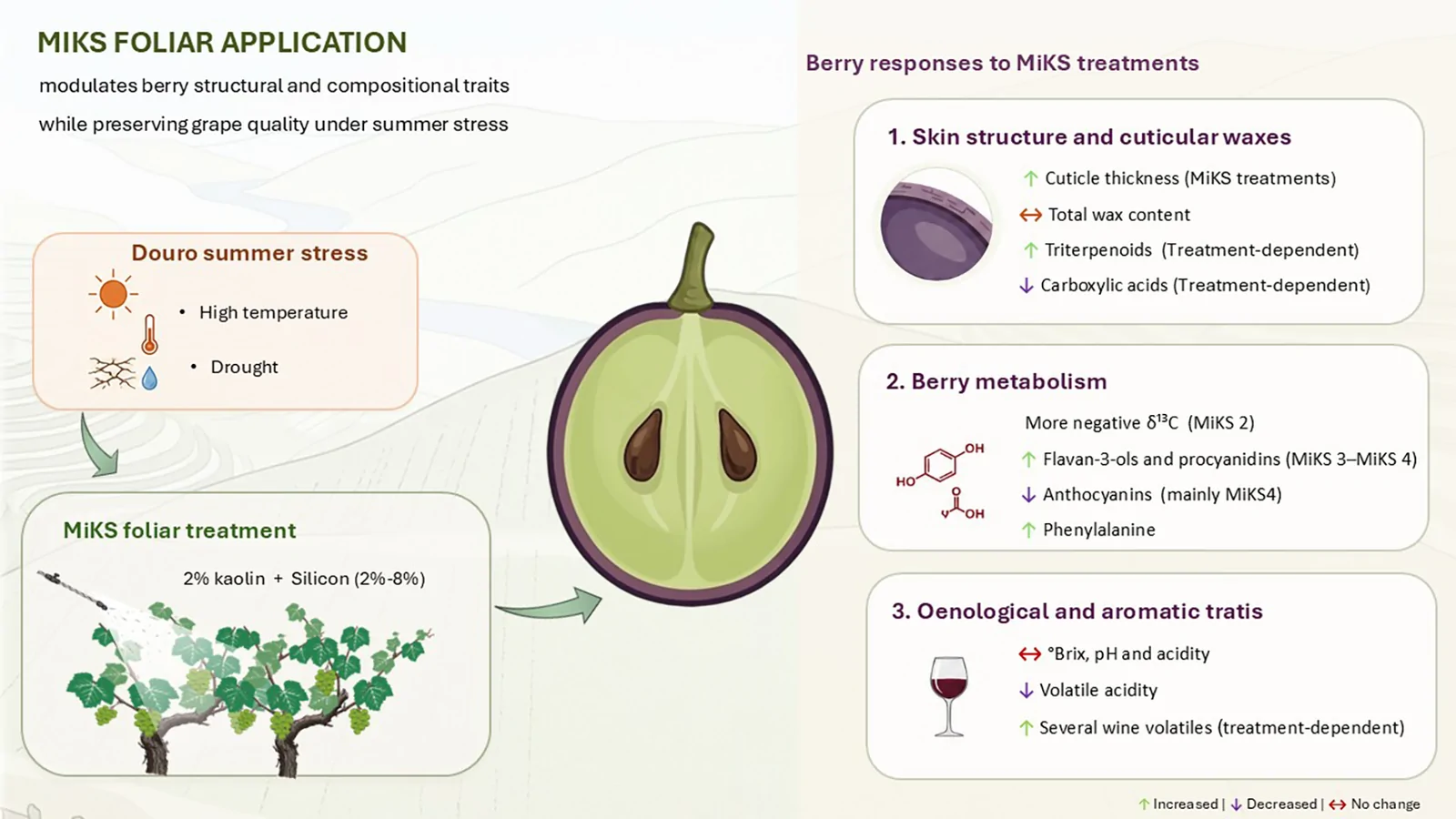

## 1 Introduction

The Douro Demarcated Region (DDR), inscribed by UNESCO as a World Heritage site since 2001, represents one of the most emblematic landscapes of Mediterranean viticulture. Despite a long history of adaptation to high temperatures, limited water availability, and the challenges of steep-slope viticulture on schist soils, recent shifts in regional climate patterns are imposing unprecedented pressure on vine performance (IVDP, 2012; Costa, 2018). Climate projections for the region indicate a progressive increase in mean annual temperatures and a reduction in precipitation, particularly during the ripening, thereby intensifying water and heat stresses (Santos et al., 2020; Droulia and Charalampopoulos, 2021; Fraga et al., 2023). These climatic conditions accelerate the phenological cycle, advancing stages such as budburst, flowering, and shifting ripening and harvests toward warmer periods (Alikadic et al., 2019; Merrill et al., 2020; Santos et al., 2020).

These environmental constraints directly affect grapevine physiological performance, disrupting carbon allocation, oxidative balance, and primary and secondary metabolism. As a result, berry development and composition are altered, with changes in sugar accumulation, organic acid degradation, and phenolic metabolism (Carvalho et al., 2015; Rogiers et al., 2022; Cataldo et al., 2023; Ferrandino et al., 2023; Conde et al., 2024). Although moderate temperature increases can accelerate phenology and sugar accumulation, prolonged heat exposure promotes organic acid degradation and disrupts anthocyanin biosynthesis and stability (Rogiers et al., 2022; Cataldo et al., 2023; Zha et al., 2024). Combined with water deficit, these conditions result in berries with higher sugar concentrations, reduced acidity, imbalanced phenolic composition, altered skin-to-pulp ratio, and, under severe stress, dehydration and structural damage (Rienth et al., 2021). Furthermore, intense solar and UV radiation increases the risk, of berry sunburn, compromising skin integrity, and ultimately affecting grape and oenological profile (Gambetta et al., 2021; Hewitt et al., 2023; Rustioni et al., 2023; Gianluca et al., 2025; Heinekamp et al., 2025). In this context, the berry skin plays a central role in protecting the fruit against environmental stress as the interface between the berry and its surrounding environment, it regulates water loss, protects internal tissues, and contains most of the phenolic compounds associated with colour, astringency and antioxidant capacity. The protective function of skin depends largely on the integrity of cuticle and epidermal, as well as on composition of cuticular waxes, which influence surface reflectance, permeability, and resistance to heat and dehydration. Therefore, it is essential for maintaining the functionality of berries under adverse conditions. Consequently, changes in skin structure and wax composition are often associated with plant’s adaptive responses to environmental stress. In viticulture, protective foliar treatments have emerged as sustainable strategies to mitigate the adverse effects of summer stress (Frioni et al., 2019; Dinis et al., 2018; Brito et al; Sangiorgi (Dinis et al., 2018; Brito et al., 2019; Frioni et al., 2019; Sangiorgio et al., 2024). Among these, Kl has been widely investigated due to its ability to form a reflective particle film that reduces leaf and berry temperature, improves plant water status, and preserves physiological performance and fruit quality (Bernardo et al., 2018; Dinis et al., 2018; Frioni et al., 2019; Terán et al., 2024). In fact, the positive effects of Kl on plant physiology, metabolism, and yield, have been reported in different crops (Dinis et al., 2018; Frioni et al., 2019; Petoumenou, 2023; Teker, 2023).

Silicon (Si) although not considered an essential element for grapevines, has also shown considerable potential to enhance plant tolerance to abiotic stress, by reinforcing cell walls, reducing water loss, activating the antioxidant defense system, and regulating genes associated with oxidative stress responses and phenolic biosynthesis (dos Santos Sarah et al., 2021; Hassan et al., 2024; Narula and Chaudhry, 2025). Together, the complementary modes of action suggest that combining Kl and Si may provide enhanced protection against environmental stress while contributing to maintenance of berry development and composition (Pereira et al., 2024).

Although the beneficial effects of Kl and Si on grapevine physiology have been widely documented, including studies from our research group demonstrating improvements in photosynthetic activity, plant water status, and antioxidant capacity (Dinis et al., 2018; Pereira et al., 2025; Monteiro et al., 2026), their combined effects on berry structural traits, epicuticular waxes, metabolic composition, and ripening dynamics remain poorly understood. Addressing this knowledge gap is essential to better understand how integrated foliar treatments influence berry quality and to support the development of sustainable adaptation strategies for Mediterranean viticulture.

Within the framework of sustainable viticulture and global climate adaptation priorities, as outlined in the United Nations 2030 Agenda, particularly Sustainable Development Goal 13 (Climate Action), it is urgent to develop effective strategies that mitigate climate-related stresses while preserving grape quality. In this context, it is essential to understand how berries directly respond to foliar treatments in order to design targeted and efficient management practices.

Therefore, this study evaluated the effects of a combined foliar application of 2% Kl with increasing Si concentrations (2%, 4%, 6%, and 8%) on berries of Touriga Nacional variety, one of the emblematic red grape varieties of the Douro region. Three dimensions were considered: (i) the biochemical impact on primary and secondary berry metabolites (sugars, organic acids, and phenolic compounds); (ii) the anatomical characterization of the skin and epicuticular waxes (thickness, organization and composition); and (iii) the modulation of ripening dynamics and the final grape composition. The aim of this study was to evaluate the response of grape berries to different Kl-Si foliar formulations under summer stress conditions and to assess their potential contribution to the sustainability of Douro viticulture under climate change.

## 2 Materials and methods

### 2.1 Study site and plant material

The study was carried out during the summer months (from June to September) of 2023 and 2024 growing seasons, in a commercial vineyard at “Quinta do Bomfim”, Pinhão, situated in the Cima-Corgo sub-region of the DDR, in Portugal (41°11′21,28″N. 7°32′27.40″W). The experimental trial was set up in a Touriga Nacional vineyard, planted in 2015 and grafted onto 110 Richter (R110) rootstock. The vines were trained using a unilateral Royat cordon system with vertical shoot positioning (VSP), and each vine was pruned to 3 to 4 spurs (6 to 8 buds per vine). The row and vine spacings were 2.00 m and 1.00 m, respectively, and the rows were oriented from east–southeast to west-northwest. The vineyard was managed under drip irrigation, with irrigation applied once a week according to the commercial management practices of the vineyard.

### 2.2 Climatic characterization

**Figure 1:**
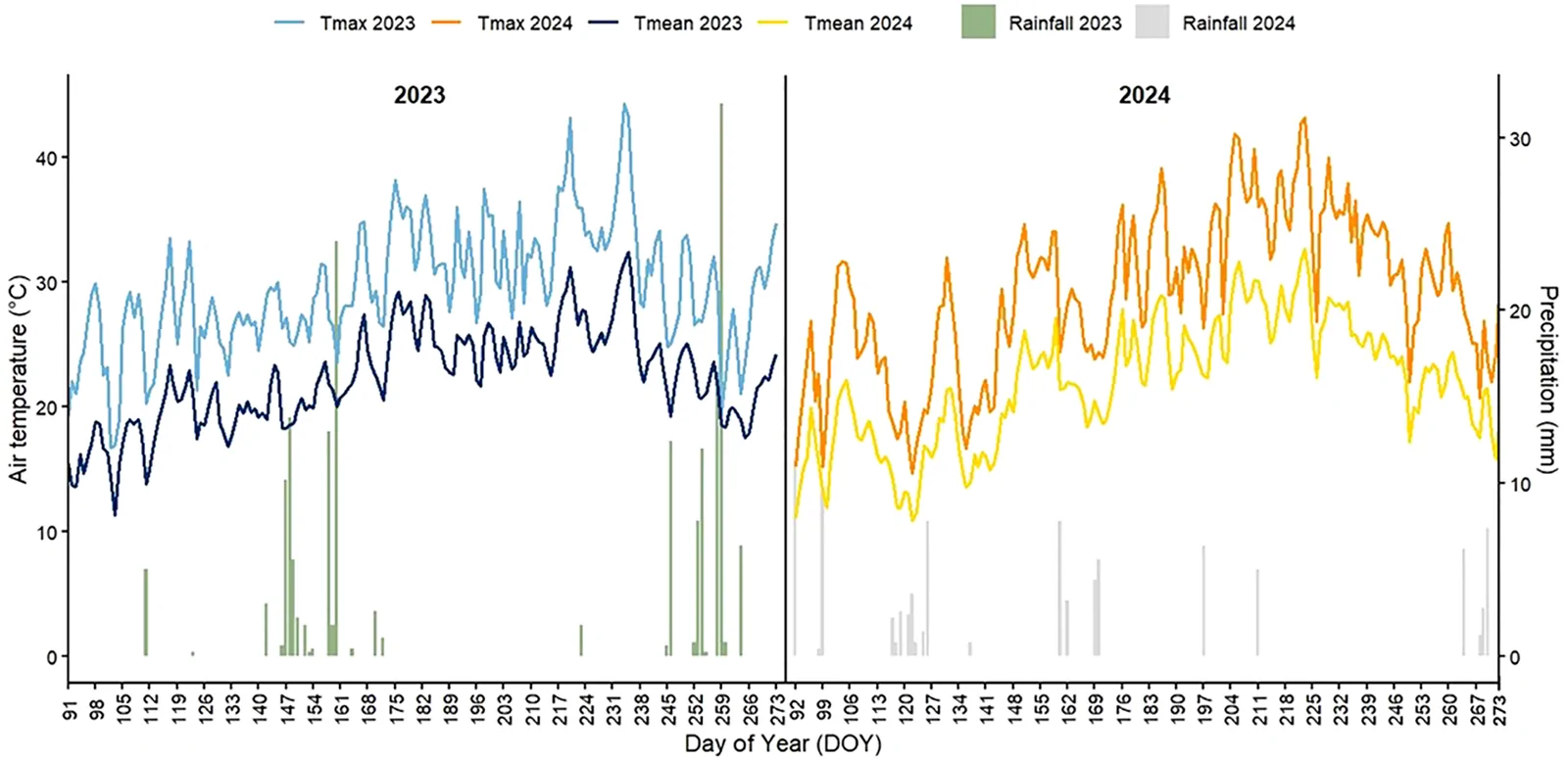
Daily mean air temperature (Tmean (°C), represented by the lines), and precipitation (mm, represented by the bars) recorded in Quinta do Bomfim, in Cima-Corgo sub-region of DDR, from April 1 (DOY 91) to September 30 (DOY 273) for the years 2023 and 2024. DOY (Day of the Year).

The vineyard was managed in accordance with the company’s standard commercial practices. Meteorological data for both growing seasons was automatically recorded by a weather station located near the experimental site. **Figure 1** shows the daily precipitation and means air temperature expressed as day of the year (DOY) for 2023 and 2024. From April 1 (DOY 91) to September 30 (DOY 273), the mean air temperature was 22.3 °C in 2023, decreasing slightly to 21.9 °C in 2024. The number of days with temperatures exceeding 35 °C increased markedly from 25 to 35, while the occurrence of extreme heat events above 40 °C increased from 4 to 6 days. Over the same period, total precipitation decreased considerably from 177.0 mm in 2023 to 93.6 mm in 2024, representing 30.3% and 18.3% of the annual totals, respectively.

**Table 1** summarizes the main bioclimatic indices and their classifications for two seasons. The Dryness Index (DI) reached 22.2 mm in 2023 and −43.4 mm in 2024, both classified as “moderately dry” (DI +1) according to Tonietto and Carbonneau (2004). These values indicate that grapevines experienced moderate water deficit conditions, which were more intensite in 2024.

**Table 1:** Bioclimatic indices [Dryness Index (DI), Huglin Index (HI), and Cold Nights Index (CI)] and their classifications for 2023 and 2024 growing seasons according Huglin (1979) and Tonietto and Carbonneau (2004).

Bioclimatic index	2023	Classification	2024	Classification
Dryness index (DI) 22.2	22.2	Moderately dry (DI +1)	-43.4	Moderately dry (DI +1)
Huglin Index (HI)	2976.2	Warm (HI +2)	3986.5	Very warm (HI +3)
Cool Night (IF)	18.6	Warm nights (CI-2)	16.9	Temperate nights (CI-1)

Similarly, the Huglin Index, an indicator of thermal accumulation during the growing season, increased from 2976.2 in 2023 to 3986.5 in 2024, corresponding to a shift from a “warm temperate climate” (HI +2) to a “very warm temperate climate” (HI +3) (Huglin, 1978; Tonietto and Carbonneau, 2004). This increase suggests that heat stress was more pronounced in 2024, resulting in more demanding conditions for grapevine development.

The Cold Nights Index (CI) indicates nighttime temperature during grape ripening. Recorded values were 18.6°C in 2023 (warm nights) and 16.9°C in 2024 (mild nights). These milder nighttime temperatures in 2024 provided thermal relief, partially mitigating daytime heat stress (Tonietto and Carbonneau, 2004).

### 2.3 Foliar treatments and application

A randomized complete block design was adopted, comprising three blocks separated by five to six untreated vine rows to minimize spray drift during foliar applications. Within each block, the four foliar treatments were assigned to individual vineyard rows, each containing 20 grapevines and representing one experimental unit. Consequently, each treatment was replicated three times (n = 3), resulting in a total of 240 grapevines.

**Figure 2:**
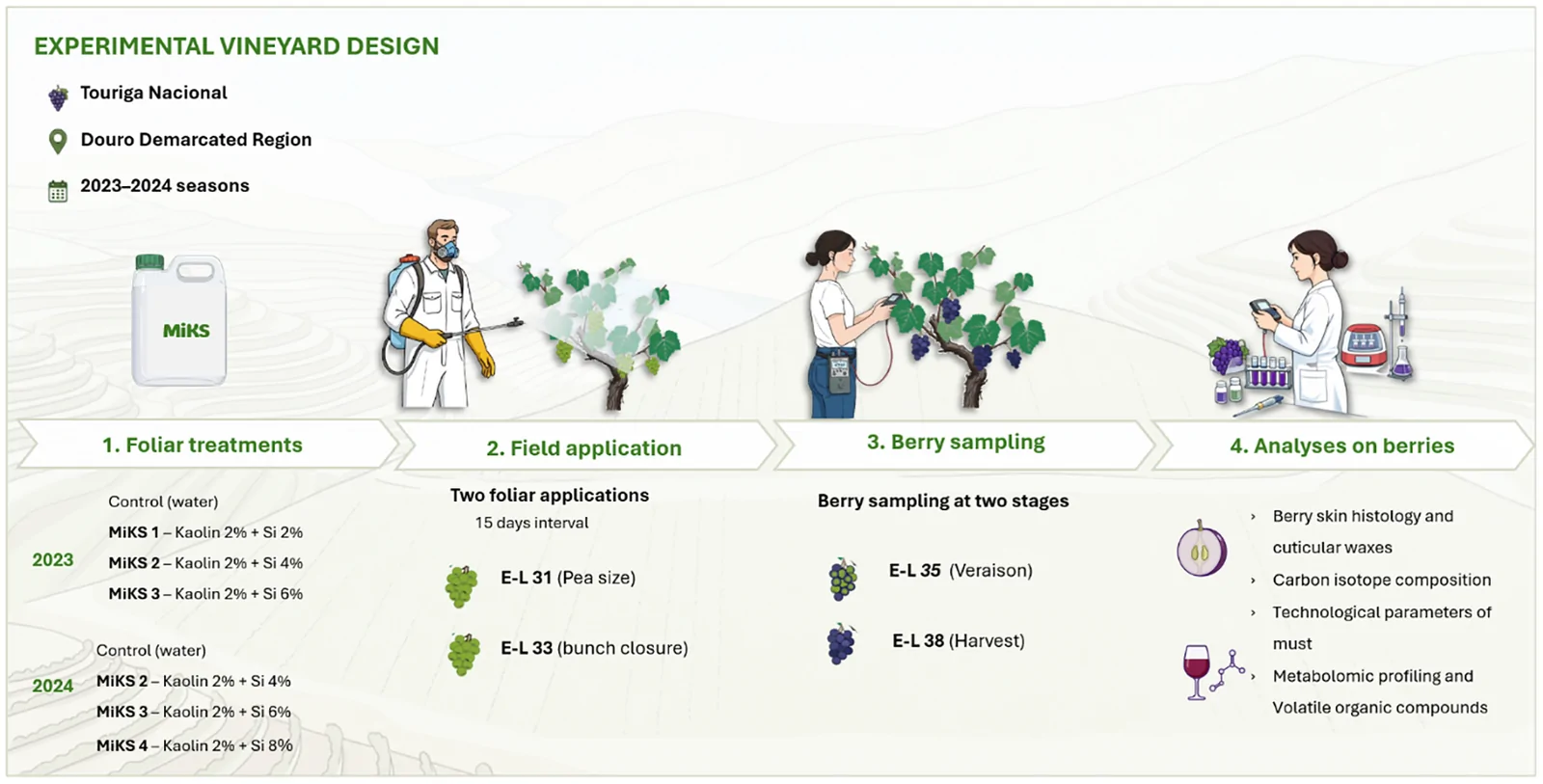
Timeline of foliar applications treatments during the 2023 and 2024 growing seasons in grapevine variety Touriga Nacional, Cima-Corgo sub-region, Douro Demarcated Region (DDR), Portugal.

In 2023, four foliar treatments (w/v in water) were applied: (i) Control (CTRL, foliar spraying with water); (ii) MiKS 1 (2% kaolin + 2% Si); (iii) MiKS 2 (2% kaolin + 4% Si), and (iv) MiKS 3 (2% kaolin + 6% Si), based on proportions previously reported (Dinis et al., 2024). In the second year (2024), the experimental design was adjusted to further explore the effects of higher Si concentrations. Accordingly, MiKS 1 was replaced by MiKS 4 (2% kaolin + 8% Si), while the other treatments (CTRL, MiKS 2, and MiKS 3) remained the same. The formulations were prepared using the following commercial products supplied by Tecniferti, (Portugal); Humigel Kaolin and HumigelPlus A. The HumigelPlus contains Si (6% w/w) soluble silicon (SiO_2_). Spray solutions were prepared by first diluting the Si formulation in water, then gradually adding Kl under continuous agitation to obtain a homogeneous suspension, without the use of adjuvants. All treatments were applied in the early morning when temperatures were lower, using a manual sprayer to ensure complete coverage of both sides of the canopy.Two foliar applications were performed per season: the first at pea-size (E-L31), followed by a second application 15 days later, as shown in **Figure 2**. At each sampling date, corresponding to veraison (E-L35) and harvest (E-L38), one cluster was randomly collected from each five vines within each treatment row (experimental unit). Berries collected from the five clusters constituted the representative sample of each experimental unit and were subsequently processed according to the requirements of each analytical procedure. Samples intended for histological, cuticular wax, and oenological analyses were maintained fresh at 4°C and processed shortly after collection. In contrast, berry samples designated for metabolomic and biochemical analyses were immediately frozen in liquid nitrogen, lyophilized, and stored until extraction and analysis. Port wines were produced following the traditional vinification practices adopted in the Douro Demarcated Region for Port wine production.

### 2.4 Berry skin structure and cuticular waxes analysis

#### 2.4.1 Berry skin histology

To assess berry skin structure, six grape berries per treatment/phenological stage (n=6) were randomly collected during the 2024 growing season and transversally sectioned as an equatorial region to minimize structural variability. The samples were immediately fixed in a FAA solution (formalin–acetic acid–alcohol) and subsequently dehydrated through a series of ethanol baths (70–100%). After dehydration, the samples were embedded in paraffin and sectioned into thin transverse slices (4 µm) using a rotary microtome (Leica RM2135 Leica Biosystems, Germany). The sections were mounted on glass slides and stained with 0.1% toluidine blue. The stained sections were examined using an Olympus IX51 inverted light microscope (Olympus Corporation, Japan), and tissue dimensions were quantified using Digimizer image analysis software (MedCalc Software, Ostend, Belgium).

#### 2.4.2 Cuticular wax extraction

The cuticular wax extraction was performed during the 2024 growing season following previously described methods (Trivedi et al., 2019), with slight modifications. Briefly, grape berries were quickly rinsed with distilled water to remove surface impurities prior to extraction. For each treatment, twenty-five intact berries were used (n=25 per treatment), and divided into five replicates of five berries (n=5 replicates).

Replicates were weighed and immediately immersed in 20 mL of chloroform (≥ 99%, suitable for HPLC, Sigma-Aldrich, Germany) for 30 seconds at room temperature under a fume hood. Extracts were filtered through filter paper (Filter-Lab, grade 1300/80, Ø 90mm) and left at room temperature to evaporate completely for a few days. Total wax amount was expressed as micrograms per gram of fresh mass (µg g^-1^ FM), normalized to berry fresh weight within each extraction vial.

#### 2.4.3 Wax composition analysis

Cuticular wax composition analysis was performed during the 2024 growing season. Cuticular residues obtained from cuticular wax extraction (section 2.4.2) were re-dissolved in 5 mL of chloroform, from which 100 µL aliquots (equivalent to 25 mg in 5.5 mL) were collected. An internal standard (tetradecane, 80 µL, 500 ng·mL^-1^) was added, and samples were dried under a nitrogen stream and derivatized with BSTFA: pyridine (1:1, v/v) at 70°C for 30 min to obtain trimethylsilyl TMS derivatives. Gas chromatography-mass spectrometry (GC-MS) analysis was performed using an HP5-MS column (30 m × 0.25 mm × 0.25 µm; Agilent J&W, USA) with helium as carrier gas. Samples were injected at 280°C in pulsed splitless mode. A 5977B mass spectrometer detector (Electron impact at 70 eV, scan range m/z 50–650) was used for compound identification, while quantification was carried out using a flame ionization detector (FID) at 280°C.

Compounds were identified based on reference standards and comparison with NIST mass spectral library (match factor > 700). Background contaminants identified using blanks and TraceCERT^®^ standards such as phthalates and glycerol were excluded from final area calculations. Wax compounds were quantified and expressed as µg g^-1^ FM, normalized to berry fresh weight and reported as relative abundance (%) of total cuticular wax.

### 2.5 Carbon isotope ratio (δ^13^C) analysis of grape must

The carbon isotope discrimination (δ¹³C) of grape must was determined during the 2024 growing season following the method described by (Gaudillère et al., 2002; Serrano et al., 2022) using a ThermoQuest Flash 1112 elemental analyzer coupled to a Delta-Plus isotope ratio mass spectrometer (IRMS) via a ConFlo III interface (ThermoQuest, Bremen, Germany). Aliquots of 1 µl of must was sealed in a tin capsule, oxidized to CO_2_, and separated by gas chromatography prior to isotopic analysis by mass spectrometry. δ¹³C values were calculated relative to the Vienna Pee Dee Belemnite (VPDB) standard. Nine samples per treatment (n=9) were analyzed.

### 2.6 Berry metabolites analysis

#### 2.6.1 Preparation of grape berry extracts

Lyophilized grape berry powder (40 mg), obtained from samples collected during the 2023 and 2024 growing seasons, was homogenized and extracted with methanol: water: HCl (70:30; v/v) at an initial concentration of 4 mg·mL^-1^ or further diluted as described by Luzio et al. (2021). The homogenate was centrifuged at 12000×g for 15 minutes at 4 °C, and the supernatant (fruit extract) was collected for subsequent analysis, following the procedure described by Lemos et al. (2020). All analyses were performed in triplicate.

#### 2.6.2 Phenolic composition and organic acids

Total phenolic content was determined using the Folin-Ciocalteu method, with absorbance measured at 725 nm, as previously described (Rodrigues et al., 2015). The results were expressed in milligrams of gallic acid equivalent per gram of dry weight (mg GAE·g^-1^ DW). Total flavonoids were quantified using the aluminum chloride colorimetric assay, with an absorbance reading at 510 nm, and the results were expressed as milligrams of catechin equivalents per gram of dry weight (mg CAE·g^-1^ DW).

Condensed tannins were quantified using the vanillin-HCl method, with absorbance measured at 500 nm, following the procedure described by Dambergs et al. (2012). The results were expressed as milligrams of catechin equivalent per gram of dry weight (mg CAE·g^-1^ DW). Total anthocyanin content was determined using pH difference method, and results were expressed as milligrams of cyanidin-3-glucoside equivalent per gram of dry weight (mg cyd-3-glu·g^-1^ DW).

### 2.7 Grape maturity and oenological parameters

Oenological parameters were determined for grape samples collected during the 2023 and 2024 growing seasons. Soluble solids (°Brix), pH, total acidity (g L^-1^ of tartaric acid), volatile acidity (g L^-1^ of acetic acid), and yeast assimilable nitrogen (YAN, mg N L^-1^), as well as color parameters, such as hue and color intensity (Abs 420 nm), total phenols and total reactive phenols (TRP), were determined using Fourier Transform-infrared spectroscopy (FT-IR Multispec; TDI, Barcelona, Spain), following standard methods (European Commission, 1990), and OIV methodologies (OIV, 2003).

### 2.8 Berry metabolite profiling

Metabolic extraction and Ultra-High Performance Liquid Chromatography coupled with Mass Spectrometry (UHPLC–MS) analysis were performed on berry samples collected in the 2024 growing season. Metabolites were extracted from 50 mg of lyophilized and finely ground berry sample using 1 mL of methanol/water solution (80:20, v/v). Samples were sonicated in an ice-cooled ultrasonic bath containing ice-cold water for 30 minutes in a cold room and subsequently maintained at lower temperature overnight to enhance metabolite extraction. Following centrifugation (16,900 × g 10 minutes, at 4°C), 600 µL of the supernatant were collected and stored at −20°C until analysis.

Before UHPLC–MS analysis, extracts were diluted (1:5) in H_2_O (95:5, v/v) containing 0.1% formic acid. Chromatographic separation was performed using an ACQUITY™ Ultra Performance Liquid Chromatography (UPLC) system coupled to a Xevo TQ-S Cronos triple quadrupole mass spectrometer (Waters, Milford, MA, USA), equipped with an electrospray ionization (ESI) source and operated via MassLynx v4.1 software (Waters, Milford, MA, USA). Compound identification was based on a combination of retention times, mass-to-charge ratios (m/z), and UV–Vis spectra, through comparison with authentic standards, in-house purified compounds, and previously published data (Martins et al., 2020, 2021, 2025; Miliordos et al., 2022). Data acquisition was performed in selected ion monitoring (SIM) mode, with major ions (m/z) monitored for each metabolite report in **Supplementary Table 2**.

### 2.9 Identification and quantification of volatile organic compounds

The identification and semi-quantification of volatile organic compounds (VOCs) were performed using wine samples collected during the 2024 growing season. Wines were produced according to the standard vinification protocols of Symington Family Estates, following the regulations and winemaking practices established by the Instituto dos Vinhos do Douro e do Porto (IVDP) for Port wine production.

Volatile compounds were extracted using headspace solid-phase microextraction (HS-SPME) and analyzed by GC-MS. The analytical procedure was internally developed and optimized for Port wine analysis. No ethanol removal or additional sample preparation was performed prior to extraction.

Wine samples (5.0 mL) were directly placed in 20 mL glass vials containing 500 mg of sodium chloride and 50 µL of 2-dodecanol (internal standard, 40 mg L^-1^). For VOC extraction, samples were incubated at 45°C for 5 minutes, with intermittent agitation (250 rpm; 2-second on/off cycles) to promote phase equilibration. The SPME fiber (type DVB/CAR/PDMS (2 cm)) was then exposed to the headspace for 30 minutes under the same conditions. Thermal desorption of the adsorbed compounds was carried out directly in the GC injector at 250°C for 2 min in splitless mode. Separation and detection were performed using a GC-MS system coupled to a triple quadrupole mass spectrometer equipped with a DB-WAX column (30 m × 0.32 mm ID × 0.25 µm film thickness). The oven temperature program was as follows: initial temperature at 40°C (1 min), followed by heating ramps of 1.5°C min^-1^ to 100°C (1 min), then 3.5°C min^-1^ to 180°C (1 min), and finally, 15°C min^-1^ to 230°C, held for 26 min. Helium was used as the carrier gas at a constant flow rate of 2 mL min^-1^.

The GC/MS interface temperature was maintained at 230°C and the ion source at 240°C. Ionization was performed by Electron Impact (EI) at 70 eV. Data acquisition was performed in full scan mode, between 50 and 450 m z^-1^, from the beginning of the run (0 min). Compound identification was achieved by comparison with the Main Library Alliance (MainLIB) mass spectral library and corresponding Kovats retention indices (RI). Whenever available, compound identities were confirmed using certified analytical standards (STD). VOCs were semi-quantified using the internal standard (2-dodecanol) without applying compound-specific response factors, and expressed as micrograms per litre (μg L^-1^) equivalents of 2-dodecanol. Therefore, the reported VOC concentrations should be considered semiquantitative rather than absolute concentrations.

### 2.10 Statistical analysis

Statistical analyses were performed using SPSS software (version 29.0, IBM Corp., NY, USA). Differences among foliar treatments were evaluated by one-way analysis of variance (ANOVA), followed by Tukey’s *post hoc* test. Normality and homogeneity of variances were assessed using the Shapiro–Wilk and Levene’s tests, respectively. Statistical significance was set at *p <*0.05. Data are presented as the mean ± standard deviation (SD). Multivariate analysis, including principal component analysis (PCA), loading plots, and fold-change analysis, were performed using R software (version 4.3.2; R Core Team, Vienna, Austria) based on log-transformed metabolite data. PCA was performed using the complete metabolomic dataset. Log_2_ fold-change values were calculated as the logarithm base 2 of the ratio between the mean metabolite abundance of each MiKS treatment and the Control, and only metabolites showing significant treatment effects according to one-way ANOVA (p < 0.05) were included in the fold-change plot. Because the treatment structure differed between the growing seasons of 2023 and 2024, all statistical analyses were performed independently for each growing season, and no direct statistical comparisons between years were conducted.

## 3 Results

### 3.1 Anatomical characterization of the skin and cuticular waxes

**Figure 3:**
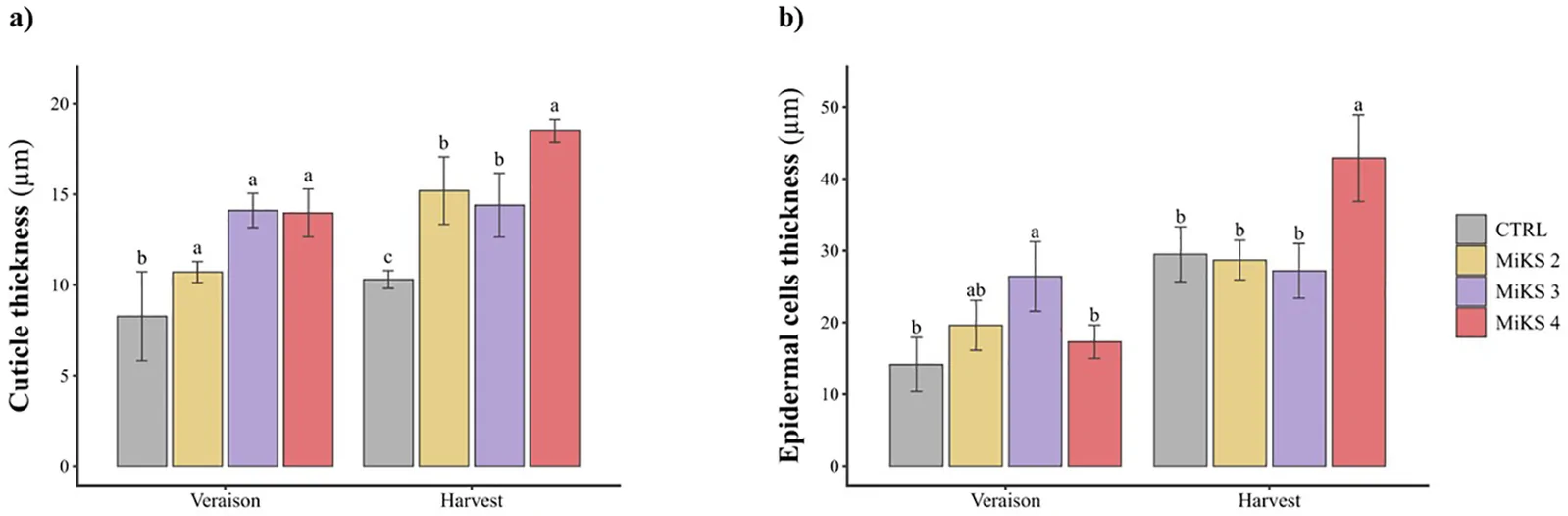
Effects of foliar applications of different kaolin (Kl) and silicon (Si) formulations (MiKS): control (CTRL water application), MiKS 2 (Kl 2% + Si 4%), MiKS 3 (Kl 2% + Si 6%) and MiKS 4 (Kl 2% + Si 8%) on berry cuticle thickness **(a)** and epidermal cell thickness **(b)** in berries of Touriga Nacional grapevine variety. Bars represent mean ± standard deviation of six berries (n=6). Different letters indicate significant differences among treatments within the same phenological stage according to Tukey’s test (*p*< 0.05).

Anatomical traits and epicuticular waxes in berry skin tissues were evaluated only during the 2024 growing season, at veraison and harvest stages. MiKS foliar treatments significantly modified berry skin structure, particularly by increasing cuticle thickness at both phenological stages (**Figures 3a, b**). Compared to the Control berries, the cuticle thickness was significantly increased by all MiKS treatments (*p <*0.001), with increases ranging from 42 to 69% at veraison and from 40 to 49% at harvest.

Epidermal cell thickness showed dependent response to berry developmental stage, with significant increases observed only in MiKS 3 (86.7%) at veraison and in MiKS 4 (45.4%) at harvest, compared with the control.

**Figure 4:**
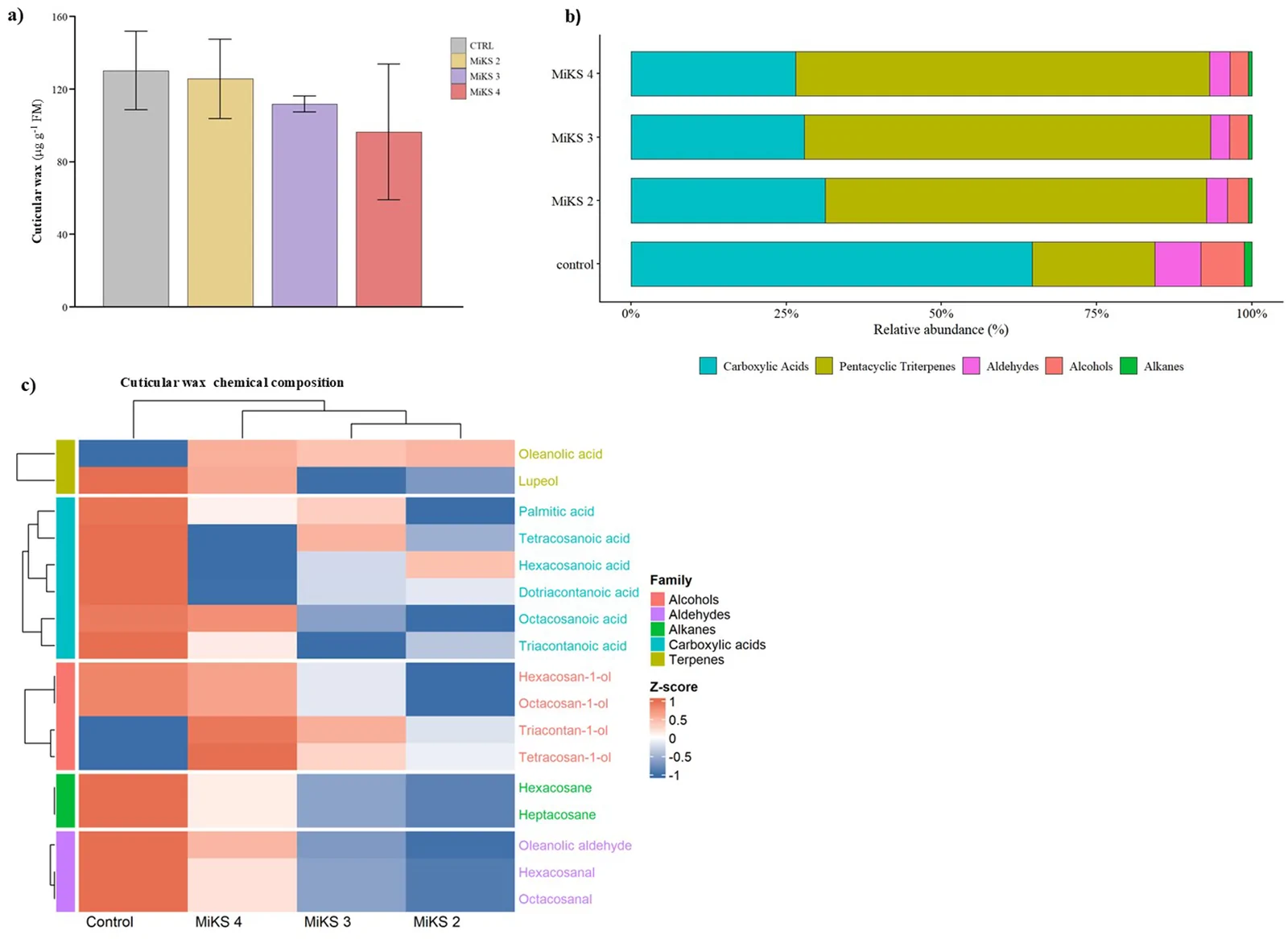
Effects of foliar applications of different kaolin (Kl) and silicon (Si) formulations (MiKS): control (CTRL water application), MiKS 2 (Kl 2% + Si 4%), MiKS 3 (Kl 2% + Si 6%) and MiKS 4 (Kl 2% + Si 8%) on total cuticular wax content **(a)**, relative abundance of compound classes identified in cuticular wax extracts **(b)**, and hierarchical clustering heatmap on z-score transformed relative abundance of individual wax constituents **(c)** in berries of grapevine variety Touriga Nacional. Bars represent mean ± standard deviation of six berries (n=5). Different letters indicate significant differences among treatments according to Tukey’s test (*p*< 0.05). Absence of letters indicates no significant differences.

Despite these anatomical modifications, MiKS treatments did not significantly affect the amount of epicuticular waxes extracted from the berries in the 2024 growing season (*p* = 0.248) (**Figure 4a**).

Additional GC–MS analyses were conducted in 2024 to evaluate the impact of MiKS foliar treatments on the cuticular wax profile of grape berries (**Figure 4b**). A total of 17 wax constituents were identified and categorized into five chemical classes: two pentacyclic triterpenoids, six carboxylic acids, four alcohols, two alkanes, and three aldehydes.

Interestingly, distinct wax signatures were observed in the Control and MiKS formulations. Pentacyclic triterpenoids were the predominant class in all MiKS treatments, accounting for 61.4-66.8% of total wax content. This was followed by carboxylic acids (26.5-31.3%), while alcohols contributed only 3-8%, depending on treatment (**Figure 4b**). Minor classes, including alkanes and aldehydes, collectively contributed less than 2% of total wax content. On average, carboxylic acids predominated in Control berries (64.6% of total wax), followed by pentacyclic triterpenes (19.8%), aldehydes (7.4%), and alkanes (1.2%). To further investigate treatments differences in wax composition, hierarchical clustering based on z-score transformation relative abundance revealed two main clusters. These clusters clearly separated the Control berries from MiKS 2, MiKS 3 and MiKS 4, which clustered together and showed similar wax profiles (**Figure 4c**). Analysis of individual wax constituents indicated that oleanolic acid was major contributor to the separation among treatments, showing the strongest effect among all quantified compounds (F=46.96; p<0.001). Its relative abundance increased from 1.28% in the Control berries to 10.6% in MiKS treated berries. Lupeol also showed a significant, although much smaller, treatment effect (F=4.77; p=0.014), with a slight decrease in relative abundance compared with the Control. In contrast, none of the remaining individual carboxylic acids, alcohols, alkanes, and aldehydes differed significantly among treatments (**Supplementary Table 1**).

### 3.2 MiKS treatments affect berry phenolic metabolism and organic acids dynamics and water status

Foliar treatments significantly influenced metabolic composition of Touriga Nacional berries, with responses varying according to growing season and phenological stage (**Table 2**). In 2023, treatments effects on phenolic composition were evident at veraison. Total phenol content was significantly higher in MiKS 2 (46.6%) compared to Control, whereas MiKS 1 and MiKS 3 did not differ significantly from Control berries. Flavonoid content decreased by 11% and 23% in berries treated with MiKS 1 and MiKS 3, respectively, while no significant differences were observed in MiKS2. Tannin content was significantly reduced in all MiKS treatments, ranging from 14 to 19% relative to Control. Anthocyanins content increased by approximately 18% in MiKS 3 and decreased by 32% in MiKS 1 relative to the Control and MiKS 2. These responses in phenolic metabolism were accompanied by organic shifts, with malic acid decreasing significantly 24 to 27% across all MiKS treatments, while tartaric acid no significant changes. However, the effects of treatment on phenolic metabolism were less pronounced at harvest. Flavonoid content decreased by 29.4% and 38.5% in MiKS 2 and MiKS 3, respectively, compared to the Control berries. Likewise, tannin reductions became progressively greater with increasing Si concentration, ranging from 30.9% in MiKS 1 to 53.7% in MiKS 3. Anthocyanin content also decreased across all treatments, with reductions ranging from 37% to 49%, compared to Control. Malic acid displayed a divergent response: it increased in MiKS 1 and MiKS 3 but decreased in MiKS 2. Tartaric acid, meanwhile, was not significantly affected by the treatments. The experimental design in 2024 included the MiKS 4 formulation, which revealed a differential metabolic response in the berries. At veraison, MiKS 4 showed a significant increase in total phenols (23%), flavonoids (25%), and tannins (23%) relative to the Control. By contrast, MiKS 2 showed the highest levels of tannins (41.2%) and anthocyanin accumulation (58%). Changes in the levels of organic acids were also evident. Malic acid content increased across the MiKS treatments, particularly in MiKS 4 (by 29%), while tartaric acid content decreased significantly in MiKS 2 (48%) and MiKS 4 (42%), compared to Control. At harvest, the differences between the MiKS treatments were most evident in terms of tannin content, which was higher in MiKS 2 (34% compared to Control). The malic acid content was significantly lower in MiKS 4 compared to all other treatments. In contrast, tartaric acid concentrations were significantly higher in MiKS 4 (32%) than in Control. MiKS 2 and MiKS 3 showed no significant differences.

**Table 2:** Effects of foliar applications of different kaolin (Kl) and silicon (Si) formulations (MiKS): control (CTRL, water application), MiKS 1 (Kl 2%+Si 2%), MiKS 2 (Kl 2%+Si 4%), MiKS 3 (Kl 2%+Si 6%) and MiKS 4 (Kl 2%+Si 8%) on primary and secondary metabolites in berries grapevine variety Touriga Nacional at veraison and harvest during the 2023 and 2024 growing seasons.

Season	Phenological	Foliar	Phenols	Flavonoids	Tannins	Anthocyanins	Malic acid	Tartaric acid
	Stage	Treatment	(mg GAE g^-1^ DW)	(mg CAE g^-1^ DW)	(mg CAE g^-1^ DW)	(mg cyd-3-glu·g^-1^ DW).)	(g L^-1^)	(g L^-1^)
2023	Veraison	Control	28.3 ± 2.30 b	33.8 ± 0.993 a	20.7 ± 1.35 a	2.49 ± 0.145 b	4.18 ± 0.117 a	2.49 ± 0.456
		MiKS 1	21.4 ± 1.75 b	30.1 ± 0.937 b	16.7 ± 1.90 b	1.69 ± 0.045 c	3.03 ± 0.258 b	3.86 ± 0.796
		MiKS 2	41.5 ± 1.89 a	33.7 ± 2.13 a	17.3 ± 1.54 b	2.51 ± 0.187 b	3.12 ± 0.198 b	3.04± 0.462
		MiKS 3	27.2 ± 2.54 b	26.1 ± 2.09 c	17.7 ± 1.03 b	2.94 ± 0.274 a	3.18 ± 0.144 b	2.88 ± 0.484
		*p*-value	<0.001	<0.001	0.011	<0.001	<0.001	0.090
	Harvest	Control	31.0 ± 2.17 ab	30.6 ± 0.952 a	24.2 ± 2.08 a	3.99 ± 0.370 a	0.713 ± 0.135 bc	3.04 ± 0.377
		MiKS 1	30.8 ± 0.651 ab	30.9 ± 0.779 a	16.7 ± 1.84 b	2.48 ± 0.061 b	0.925 ± 0.045 a	2.68 ± 0.248
		MiKS 2	28.2 ± 1.12 b	21.6 ± 1.90 b	12.4 ± 1.35 c	2.03 ± 0.035 c	0.605 ± 0.015 c	2.93 ± 0.690
		MiKS 3	32.3 ± 1.38 a	18.8 ± 2.37 b	11.2 ± 0.884 c	2.35 ± 0.043 bc	0.868 ± 0.032 ab	2.70 ± 0.158
		*p*-value	0.011	<0.001	<0.001	<0.001	0.003	0.681
2024	Veraison	Control	29.6 ± 3.07 b	23.2 ± 2.40 c	14.8 ± 0.51 c	1.84 ± 0.258 c	3.38 ± 0.206 c	4.01 ± 0.405 a
		MiKS 2	28.6 ± 4.26 b	24.9 ± 0.920 bc	20.9 ± 0.700 a	2.90 ± 0.153 a	3.97 ± 0.104 b	2.08 ± 0.263 b
		MiKS 3	30.9 ± 1.14 ab	27.8 ± 2.16 ab	14.3 ± 1.23 c	1.55 ± 0.153 c	3.59 ± 0.083 c	3.09 ± 0.590 ab
		MiKS 4	36.4 ± 3.23 a	29.0 ± 1.21 a	18.2 ± 1.75 b	2.00 ± 0.085 b	4.36 ± 0.050 a	2.34 ± 0.312 b
		*p*-value	0.019	0.002	<0.001	<0.001	<0.001	0.014
	Harvest	Control	26.6 ± 1.29	21.2 ± 2.59	10.5 ± 0.297 bc	2.55 ± 0.601	0.923 ± 0.083 a	2.28 ± 0.171 b
		MiKS 2	28.6 ± 3.33	21.1 ± 0.277	14.1 ± 1.07 a	2.51 ± 0.168	0.770 ± 0.050 a	2.68 ± 0.203 ab
		MiKS 3	28.9 ± 0.590	23.1 ± 0.544	10.2 ± 0.524 c	2.32 ± 0.121	0.870 ± 0.157 a	2.54 ± 0.301 ab
		MiKS 4	28.2 ± 3.16	21.0 ± 2.36	11.8 ± 0.625 b	2.33 ± 0.128	0.465 ± 0.085 b	3.02 ± 0.215 a
		*p*-value	0.576	0.051	<0.001	0.676	0.002	0.024

Values represent mean ± standard deviation of six replicates (n=6). Different letters indicate significant differences among treatments within the same phenological stage according to Tukey’s test (*p*< 0.05). The absence of letters indicates no significant differences.

**Figure 5:**
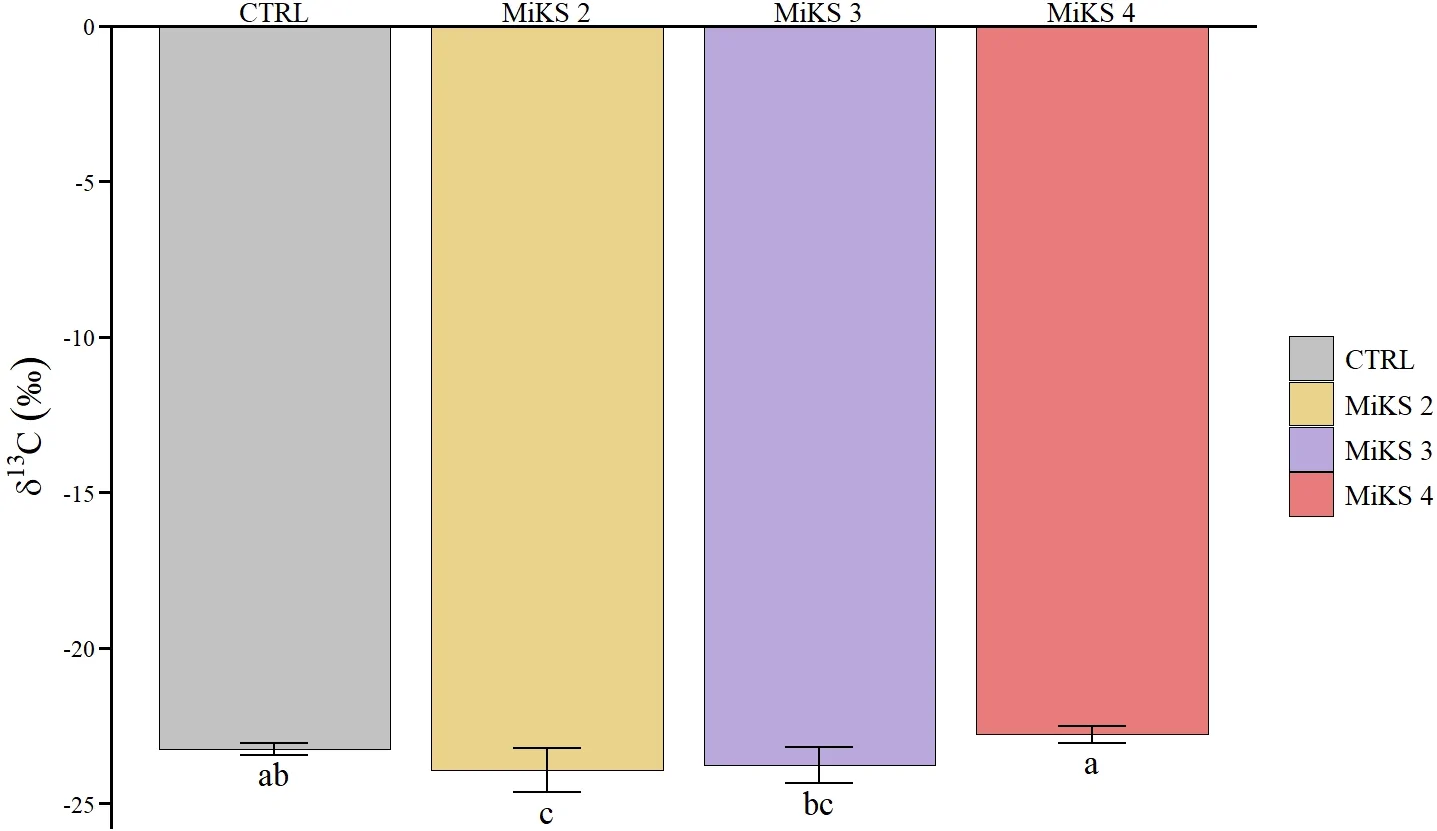
Effects of foliar applications of different kaolin (Kl) and silicon (Si) formulations (MiKS): control (CTRL, water application), MiKS 2 (Kl 2%+Si 4%); MiKS 3 (Kl 2%+Si 6%) and MiKS 4 (Kl 2%+Si 8%) on Carbon isotope ratio (δ¹³C, ‰) determined in must samples of berries from the grapevine variety Touriga Nacional, at harvest during 2024 growing season. Bars represent mean ± standard deviation of six replicates (n=9). Different letters indicate significant differences among treatments within the same phenological stage according to Tukey’s test (*p*< 0.05). The absence of letters indicates no significant differences.

Additionally, δ¹³C analysis of must samples at harvest during the 2024 growing season revealed significant differences among treatments (*p <*0.001) (**Figure 5**). MiKS 2 treatments exhibited significantly more negative δ¹³C values than the Control, indicating a 2.9% reduction.

### 3.3 Ripening dynamics and final grape quality

Overall, foliar MiKS applications had no significant effect on must technological parameters, including berry weight (g), soluble solids (°Brix), pH, and total acidity across all sampling dates or growing seasons (**Table 3**). Similarly, polyphenolic and color-related parameters, such as Hue, total color density (TCD), total phenolics and total red pigments (TRP), were not significantly influenced by foliar treatments. In contrast, volatile acidity showed an overall decreasing trend under MiKS applications, with reductions ranging from 19% to 34% in 2023 and from 18 to 45% in 2024, except for MiKS 4 which showed a slight increase of 23% in volatile acidity relative to the Control on 13 September 2024.

**Table 3:** Effects of foliar application of different kaolin (Kl) and silicon (Si) formulations (MiKS): control (CTRL, water application), MiKS 1 (Kl 2%+Si 2%); MiKS 2 (Kl 2%+Si 4%); MiKS 3 (Kl 2%+Si 6%) and MiKS 4 (Kl 2%+Si 8%) on must composition of grapevine variety Touriga Nacional.

Season	Date	Foliar Treatment	Berry weight	°Brix	pH	Total acidity	Volatile acidity	YAN	Hue	TCD	Total phenols	TRP
2023	21 August	CTRL	68.9 ± 9.01	28.0 ± 1.57	3.9 ± 0.137	4.3 ± 0.206	0.390 ± 0.030 a	287.4 ± 7.30 a	1.16 ± 0.060	6.95 ± 1.58	24.3 ± 5.58	6.86 ± 1.96
		MiKS 1	70.0 ± 10.4	26.9 ± 0.808	3.8 ± 0.049	4.5 ± 0.156	0.257 ± 0.049 b	273.2 ± 24.1 a	1.09 ± 0.110	7.67 ± 1.78	27.1 ± 5.30	7.86 ± 2.35
		MiKS 2	71.5 ± 7.7	27.1 ± 1.04	3.8 ± 0.052	4.6 ± 0.166	0.265 ± 0.015 b	292.7 ± 11.1 a	1.07 ± 0.100	7.82 ± 1.39	26.9 ± 3.99	8.11 ± 2.17
		MiKS 3	71.27 ± 5.9	27.6 ± 0.173	3.9 ± 0.090	4.5 ± 0.168	0.315 ± 0.015 ab	211.2 ± 33.5 b	0.96 ± 0.031	9.17 ± 0.741	30.0 ± 2.71	10.4 ± 0.710
		*p*-value	0.979	0.573	0.699	0.535	0.002	0.006	0.086	0.350	0.526	0.215
	6 September	CTRL	69.1 ± 17.2	30.4 ± 3.40	4.00 ± 0.079	4.79 ± 0.171	0.765 ± 0.015 a	414.4 ± 41.3 a	1.05 ± 0.129	6.90 ± 0.677	26.0 ± 3.75	7.13 ± 1.76
		MiKS 1	55.3 ± 9.22	32.7 ± 4.11	3.9 ± 0.050	4.90 ± 0.006	0.620 ± 0.040 b	421.4 ± 46.1 a	1.10 ± 0.059	8.11 ± 1.16	29.8 ± 4.81	6.68 ± 0.356
		MiKS 2	66.0 ± 4.19	30.7 ± 1.40	4.0 ± 0.097	4.85 ± 0.111	0.575 ± 0.025 b	313.3 ± 24.2 b	0.985 ± 0.071	8.45 ± 1.01	27.8 ± 2.23	8.89 ± 1.65
		MiKS 3	56.5 ± 2.62	31.8 ± 1.67	4.0 ± 0.122	4.78 ± 0.330	0.653 ± 0.084 ab	307.9 ± 36.4 b	0.985 ± 0.069	8.79 ± 1.04	31.9 ± 0.69	9.24 ± 1.71
		*p*-value	0.313	0.749	0.896	0.867	0.007	0.008	0.331	0.181	0.223	0.168
2024	30 August	CTRL	85.6 ± 4.51	22.6 ± 0.872	3.84 ± 0.032	4.10 ± 0.164	0.240 ± 0.040 a	308.5 ± 40.0	1.66 ± 0.084	2.80 ± 0.117	12.0 ± 1.13	1.40 ± 0.065
		MiKS 2	90.0 ± 1.73 a	22.3 ± 0.200	3.74 ± 0.095	4.32 ± 0.303	0.133 ± 0.006 b	270.6 ± 50.1	1.61 ± 0.058	2.79 ± 0.101	11.9 ± 1.12	1.39 ± 0.066
		MiKS 3	89.0 ± 3.46	23.1 ± 0.289	3.83 ± 0.122	4.19 ± 0.386	0.165 ± 0.025 b	309.0 ± 88.6	1.56 ± 0.093	2.97 ± 0.126	12.3 ± 0.521	1.63 ± 0.222
		MiKS 4	84.3 ± 1.53	23.3 ± 0.551	3.84 ± 0.020	4.18 ± 0.080	0.170 ± 0.020 b	295.5 ± 54.5	1.56 ± 0.094	3.25 ± 0.624	14.5 ± 1.25	1.70 ± 0.427
		*p*-value	0.154	0.200	0.317	0.775	0.006	0.849	0.442	0.347	0.052	0.346
	13 September	CTRL	93.6 ± 3.79	22.7 ± 0.360	3.90 ± 0.060	3.53 ± 0.100	0.100 ± 0.020 ab	239.1 ± 42.4	1.69 ± 0.154	3.15 ± 0.435	0.111 ± 0.018	1.36 ± 0.318
		MiKS 2	91.6 ± 3.21	23.7 ± 0.929	3.85 ± 0.040	3.84 ± 0.157	0.065 ± 0.005 ab	205.6 ± 24.7	1.55 ± 0.194	2.88 ± 0.190	0.118 ± 0.024	0.995 ± 0.922
		MiKS 3	93.0 ± 2	22.5 ± 0.404	3.89 ± 0.120	3.64 ± 0.193	0.055 ± 0.015 b	306.0 ± 70.1	1.62 ± 0.092	2.80 ± 0.294	0.115 ± 0.006	0.506 ± 0.751
		MiKS 4	90.3 ± 4.16	22.9 ± 0.751	3.95 ± 0.052	3.54 ± 0.046	0.123 ± 0.038 a	299.7 ± 73.6	1.63 ± 0.098	3.08 ± 0.215	0.120 ± 0.016	1.02 ± 0.870
		*p*-value	0.650	0.110	0.490	0.079	0.021	0.164	0.700	0.483	0.923	0.602
	16 September	CTRL	91.6 ± 7.09	23.2 ± 0.945	4.11 ± 0.117	3.55 ± 0.165	0.320 ± 0.010 a	322.7 ± 71.9	1.37 ± 0.079	4.00 ± 0.604	17.5 ± 1.32	3.70 ± 0.831
		MiKS 2	90.3 ± 1.15	23.7 ± 0.656	4.07 ± 0.036	3.71 ± 0.159	0.263 ± 0.006 b	307.0 ± 26.2	1.28 ± 0.054	3.99 ± 0.054	17.8 ± 1.42	4.11 ± 0.173
		MiKS 3	90.3 ± 9.02	23.1 ± 0.115	4.05 ± 0.114	3.57 ± 0.185	0.233 ± 0.012 b	356.2 ± 14.8	1.22 ± 0.146	4.40 ± 0.455	18.8 ± 2.76	4.85 ± 1.281
		MiKS 4	92.0 ± 2.65	23.3 ± 0.802	4.11 ± 0.031	3.40 ± 0.087	0.210 ± 0.017 b	368.8 ± 26.0	1.25 ± 0.044	4.66 ± 0.297	20.5 ± 0.530	5.20 ± 0.623
		*p*-value	0.976	0.729	0.743	0.174	<0.001	0.296	0.258	0.199	0.424	0.186

Values represent mean ± standard deviation of six replicates (n=6). Different letters indicate significant differences among treatments within the same phenological stage according to Tukey’s test (*p*< 0.05). The absence of letters indicates no significant differences. Berry weight (g), calculated from the weight of 50 berries. Soluble solids (°Brix, g sucrose per 100g), total acidity (g L^-1^ tartaric acid), volatile acidity (g L^-1^acetic acid), yeast assimilable nitrogen (YAN, mg L^-1^), Hue, (A420/A520), total colour density (TCD= A420 + A520 + A620, A.U.), total phenolics (A.U.) and total red pigment (TRP, A.U.).

In turn, yeast assimilable nitrogen (YAN) responses varied between growing seasons, with significant treatment effects observed only in 2023 (**Table 3**). Thus, at the first sampling date, MiKS 3 reduced YAN by 26.5% compared to the Control. At harvest MiKS 2 and MiKS 3 reduced YAN by 24.4 and 25.7%, respectively.

Foliar MiKS treatments significantly affected the berry metabolite profile (**Supplementary Table 2**). Treatment effects were mainly observed for metabolites related to amino acid, flavan-3-ol, procyanidin, and anthocyanin metabolism. MiKS 3 and MiKS 4 were associated with higher relative abundance of aromatic amino acids, including phenylalanine (M4) and tyrosine (M5), as well as flavan-3-ols and condensed tannins, namely catechin (M18), catechin gallate (M23), procyanidin B2 (M29), and procyanidin B4 (M31). By contrast, several anthocyanin derivatives, including cyanidin-3-O-galactoside (M7), petunidin-3-O-6-p-coumaroyl-glucoside (M13), malvidin-3-O-6-acetyl-glucoside (M12), and malvidin-3-O-6-p-coumaroyl-glucoside (M14), showed lower relative abundance than control berries.

**Figure 6:**
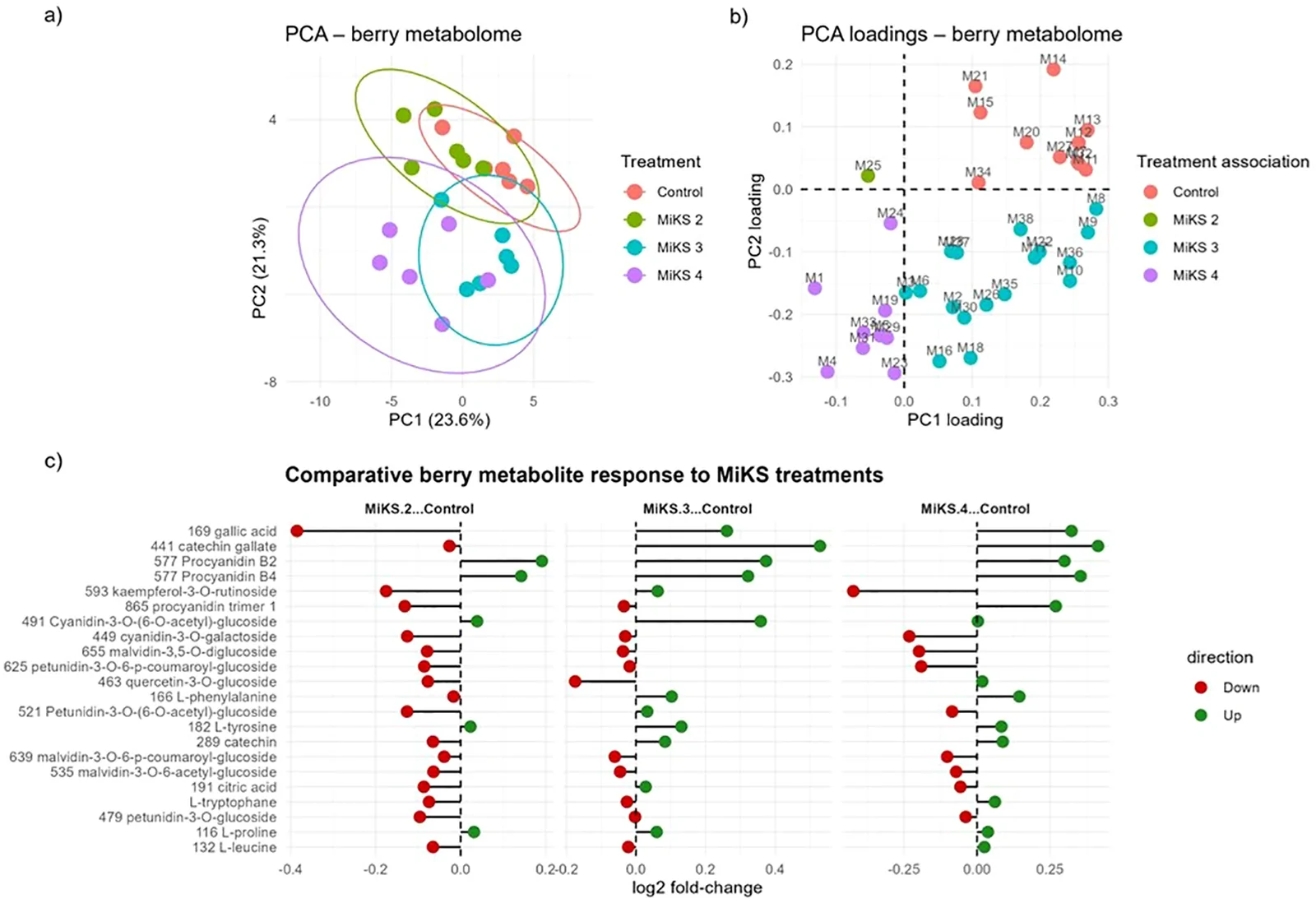
Multivariate and univariate analysis of berry metabolite responses to MiKS treatments **(a)** Principal component analysis (PCA) score plot based on the relative abundance of 38 metabolites quantified in grape berries under Control (CTRL, water), MiKS 2 (Kl 2%+Si 4%); MiKS 3 (Kl 2%+Si 6%) and MiKS 4 (Kl 2%+Si 8%) treatments. The first two principal components explained 44.9% of the total variance (PC1 = 23.6%; PC2 = 21.3%). Each point represents an individual biological replicate (n=3). **(b)** PCA loadings plot showing the contribution of individual metabolites (M1–M38) to the separation observed in the score plot. Metabolites are identified by their corresponding codes (**Supplementary Table S2** for full metabolite names). **(c)** Log2 fold-change analysis showing changes in metabolite relative abundance under MiKS treatments compared to the control. Positive/up (green) and negative/down (red) indicate increased and decreased abundance, respectively (*p*< 0.05).

A principal component analysis (PCA) was performed based on the relative abundance of 38 metabolites to explore the overall metabolic response of berries to MiKS treatments (**Figures 6a, b**). The first two principal components explained 44.9% of the total variance, with PC1 accounting for 23.6% and PC2 explaining 21.3% of the variability (**Figure 6a**). The score plot revealed a clear separation of the treatments, with the control samples clustering on the positive side of PC1, whereas MiKS 3 and MiKS 4 were displaced towards the negative PC1 axis and separated alongside PC2, suggesting distinct metabolic profiles. This separation was primarily driven by opposite contributions of anthocyanins and flavanol-related metabolites (**Figure 6b**). Positive PC1 loadings were dominated by anthocyanins derivatives, including malvidin-3-*O*-6-p-coumaroyl-glucoside (M14), petunidin-3-*O*-6-p-coumaroyl-glucoside (M13), malvidin-3-*O*-6-acetyl-glucoside (M12), and cyanidin-3-*O*-galactoside (M7), which were associated with Control and MiKS 2. In contrast, negative loadings on PC1 were mainly represented by amino acid and flavan-3-ol pathway metabolites, including phenylalanine (M4), tyrosine (M5), leucine (M2), gallic acid (M16), catechin (M18), catechin gallate (M23), and procyanidin B2 (M29), which were associated with MiKS 3 and MiKS 4. To further explore treatment effects on individual metabolites, log_2_-fold-change analysis was performed relative to the Control (**Figure 6c**). MiKS 2 induced slight increases in flavanol-related metabolites, including procyanidins B2 and B4, while reducing the several anthocyanin derivatives, particularly petunidin-3-*O*-6-p-coumaroyl-glucoside, petunidin-3-*O*-glucoside, cyanidin-3-*O*-galactoside, and malvidin-3-*O*-6-acetyl-glucoside. In contrast, MiKS 3 and MiKS 4 promoted the accumulation of flavan-3-ols and tannins, namely catechin, catechin gallate, procyanidin B2, and procyanidin B4. Similarly, amino acids and phenolic related metabolites, such as phenylalanine, tyrosine, and gallic acid increased while anthocyanins, including cyanidin-3-*O*-galactoside and petunidin-derived decreased relative to Control.

The influence of MiKS foliar application extends to the volatile profile of the resulting Port wines. VOCs analyses revealed a complex volatile composition, with 76 compounds identified, belonging to multiple chemical classes and contributing to overall aromatic complexity of the wines. Esters represent the dominant class, accounting for 40.8% of the total VOCs detected, followed by alcohols (18.4%), whereas 27.6% of the compounds remained unidentified. Minor classes included ketones (5.3%), terpenes (2.6%), carboxylic acids (2.6%), aldehyde (1.3%), and furans (1.3%) (**Supplementary Table 3**).

**Figure 7:**
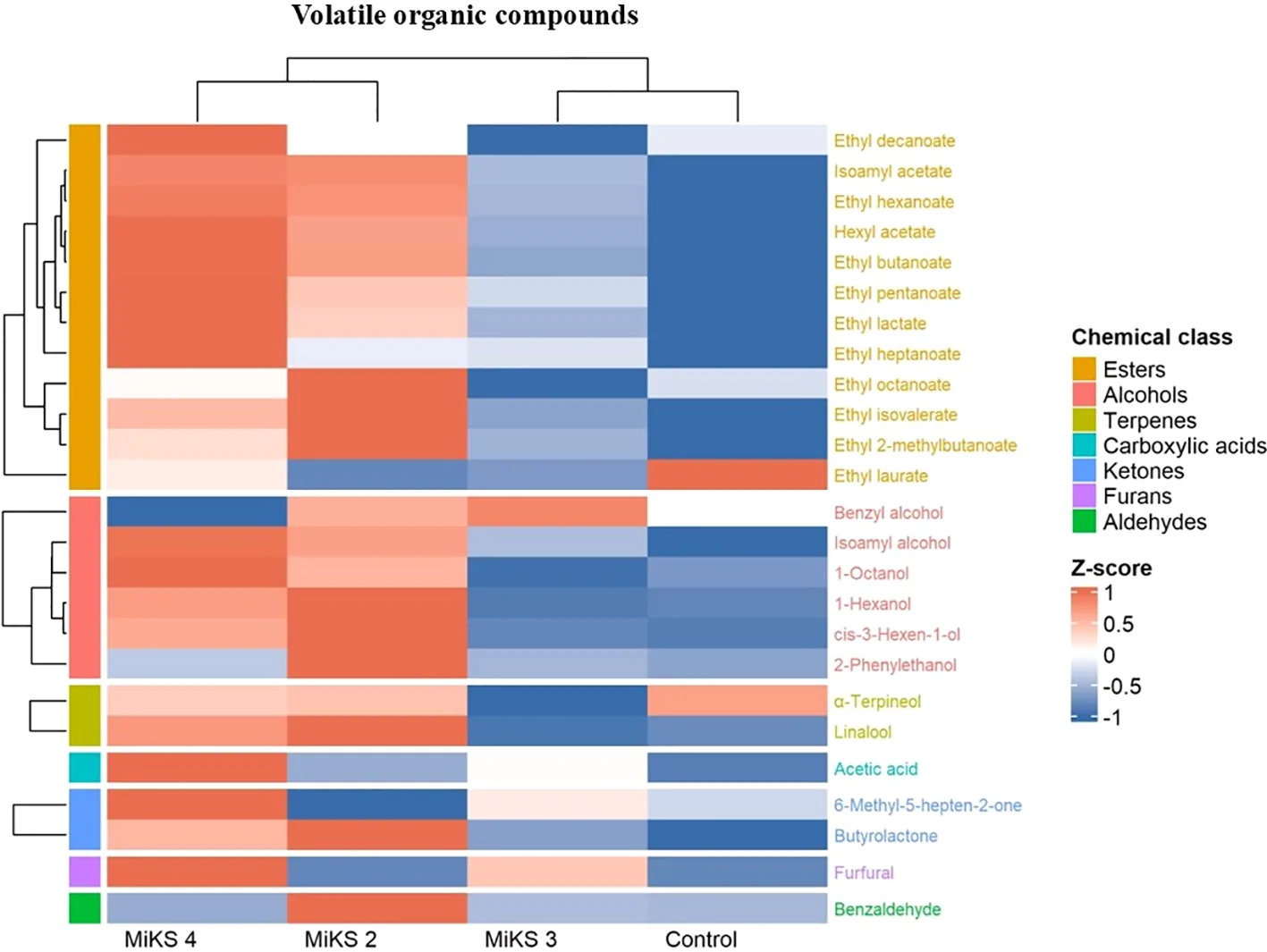
Heatmap representation of VOC profiles of wines obtained under different foliar treatments: Control, MiKS 2 (Kl 2%+Si 4%); MiKS 3 (Kl 2%+Si 6%) and MiKS 4 (Kl 2%+Si 8%)) from Touriga Nacional variety. Values correspond to row-wise Z-score normalized VOC/internal standard area ratios. Red and blue colors indicate values above and below the row mean, respectively based on row-wise Z-scores. VOCs are grouped according to their chemical class.

From these, 25 representative VOCs were selected for semi-quantitative analysis based on chromatographic resolution, abundance, and sensory relevance (**Table 4**). Treatment differences in VOC accumulation are further illustrated in the heatmap (**Figure 7**), generated using row-wise Z-score normalization. Esters were the chemical class most representative of MiKS treatments. Wines produced from MiKS 2 and MiKS 4 treated vines generally exhibited higher of several fruity esters, particularly ethyl butanoate, ethyl hexanoate and isoamyl acetate.

**Table 4:** Effects of foliar application of different kaolin (Kl) and silicon (Si) formulations (MiKS): control (CTRL, water application), MiKS1 (Kl 2% + Si 2%), MiKS2 (Kl 2% + Si 4%), MiKS3 (Kl 2% + Si 6%), and MiKS4 (Kl 2% + Si 8%) on volatile organic compound (VOC) profile of wines from grapevine variety Touriga Nacional, determined by HS-SPME-GC-MS.

Chemical class	Compound	CAS	Semi-quantitative concentration (µg L^-1^) equivalents of 2-Dodecanol				p-value	Aroma descriptors	Kovats index
			Control	MiKS 2	MiKS 3	MiKS 4			
	*Internal Standard*(2-dodecanol)	10203-28-8	400	400	400	400			
Esters	Ethyl butanoate	105-54-4	66.4 ± 4.56 b	94.4 ± 6.47 a	74 ± 8.22 b	99.2 ± 1.39 a	<0.001	Apple, banana	1020
	Ethyl 2-methylbutanoate	7452-79-1	21.3 ± 1.52 c	40.3 ± 3.19 a	25.5 ± 2.70 b	31.9 ± 0.309 c	<0.001	Fruity, green apple	1035
	Ethyl isovalerate	108-64-5	38.0 ± 5.52 b	66.3 ± 5.43 a	43.2 ± 4.83 b	57.8 ± 0.886 a	<0.001	Anise, sweet, apple, currant	1049
	Isoamyl acetate	123-92-2	437.8 ± 58.1 b	782.7 ± 42.3 a	561.2 ± 81.7 b	792.1 ± 75.3 a	<0.001	Banana, fruit, sweet	1105
	Ethyl pentanoate	539-82-2	5.46± 0.839 b	7.4 ± 0.315 a	6.60± 0.685 ab	8.20± 0.687 a	0.004	Fruity, orange, mint	1115
	Ethyl hexanoate	126-66-0	1817.3 ± 166.2 b	2921.1 ± 143.1a	2197 ± 184.2 b	3003.4 ± 130.9 a	<0.001	Fruity, green apple, banana, brandy	1217
	Hexyl acetate	142-92-7	49.4 ± 4.34 c	88.9 ± 4.36 a	62.2 ± 5.57 b	96.1 ± 2.28 a	<0.001	Apple, cherry, pear, floral	1254
	Ethyl heptanoate	106-30-9	32.7 ± 7.87 b	44.4 ± 5.69 b	43.4 ± 3.21 b	64.5 ± 8.32 a	0.002	Fruity	1316
	Ethyl lactate	687-47-8	53.2 ± 3.13 c	74.9 ± 3.35 b	61.9 ± 6.22 c	88 ± 2.82 a	<0.001	Ethereal, buttery	1325
	Ethyl octanoate	106-32-1	3518.8 ± 585	4843.5 ± 1119	2733.4 ± 519.9	3778.2 ± 892.5	0.072	Brandy, fruity, grape	1420
	Ethyl decanoate	110-38-3	1994.1 ± 304.7 bc	2075.5 ± 213.2 b	1519.1 ± 120.9 c	2670.7 ± 98.9 a	<0.001	Brandy, fruity, grape	1621
	Ethyl laurate	106-33-2	92.4 ± 4.86 a	63.4 ± 5.89 b	65.1 ± 17.4 b	76.4 ± 5.55 ab	0.024	Mango	1826
	Isoamyl alcohol	123-51-3	9586.2 ± 501.8 b	13852.4 ± 556.1 a	11273.2 ± 1396.9 b	14503.3 ± 198.5 a	<0.001	Pungent, balsamic, malty, burnt	
Alcohols	1-Hexanol	111-27-3	470.2 ± 54.2 b	646.4 ± 79.9 a	463.1 ± 45.7 b	615.7 ± 20.1 a	0.005	Herbaceous, grass, woody	1342
	cis-3-Hexen-1-ol	928-96-1	9.82 ± 1.04 b	14.0 ± 1.86 a	9.96 ± 1.108 b	13.0 ± 0.729 ab	0.007	Green, bitter	1368
	1-Octanol	111-87-5	13.2 ± 1.89 b	16.5 ± 3.50 a	12.5 ± 1.13 b	18.1 ± 0.679 a	0.033	Metallic, burnt match	1546
	Benzyl alcohol	100-51-6	9.24 ± 2.57	10.5 ± 2.18	11.1 ± 4.31	6.13 ± 0.220	0.199	Fruity, walnut, bitter almond	1828
	2-Phenylethol	60-12-8	1826.1 ± 118.2 b	3159.8 ± 78.7 a	1903.8 ± 51.9 b	1989.6 ± 128.1 b	<0.001	Rose, honey	1874
Terpenes	Linalool	78-70-6	24.6 ± 4.18 b	36.7 ± 5.38 a	23.6 ± 2.61 b	34.9 ± 1.77 a	0.004	citrus, floral	1636
	α-Terpineol	98-55-5	10.6 ± 0.824 a	10.1 ± 1.11 a	5.5 ± 0.650 b	9.9 ± 0.386 a	<0.001	Lilac, floral, sweet	1670
Carboxylic acids	Acetic acid	64-19-7	88.6 ± 21.6 b	123.7 ± 38.3 b	190.4 ± 31.7 b	337.1 ± 67.3 a	<0.001	Vinegar	1428
Ketones	6-Methyl-5-hepten-2-one	110-93-0	14.4 ± 2.77 bc	10.4 ± 0.904 c	16.4 ± 1.46 b	21.4 ± 1.88 a	<0.001	Mushroom, earthy, cooked fruit	1323
	Butyrolactone	96-48-0	3.86 ± 0.210 c	6.44 ± 0.413 a	4.31 ± 0.085 c	5.70 ± 0.145 b	<0.001	Sweet, caramel	1567
Furans	Furfural	98-01-1	115.6 ± 12.8 b	115.5 ± 7.09 b	154.8 ± 23.1 a	180.1 ± 9.34 a	<0.001	Toasted almond	1432
Aldehydes	Benzaldehyde	100-52-7	46.5 ± 8.47 b	90.6 ± 13.9 a	47.1 ± 3.91 b	44.7 ± 1.76 b	<0.001	bitter almond, smoky	1476

Values are expressed as µg L^-1^ equivalents of the internal standard 2-dodecanol.

Ethyl octanoate, ethyl decanoate, and ethyl hexanoate were the most abundant esters across all treatments, with ethyl decanoate reaching its highest concentrations in MiKS 4 wines. Furthermore, hexyl acetate, ethyl heptanoate, and ethyl lactate showed increased concentrations, particularly under MiKS 4, whereas isoamyl acetate and ethyl pentanoate were more pronounced in MiKS 2 wines. Regarding alcohols, isoamyl alcohol and 1-hexanol significantly increased in wines from MiKS 2 and MiKS 4 treatments, while 2-phenylethyl alcohol reached its higher concentration in MiKS 2. In turn, terpenes exhibited a distinct response pattern, with linalool concentrations significantly enhanced in MiKS 2 and MiKS 4 compared to the control. In contrast, α-terpineol significantly decreased in MiKS 3 wines while remaining comparable among the other MiKS treatments. Among the minor VOC classes, MiKS 4 wines exhibited the highest acetic acid concentrations, whereas furfural levels significantly increased under MiKS 3 and MiKS 4 treatments. In contrast, benzaldehyde and butyrolactone were more abundant in MiKS 2 wines.

## 4 Discussion

### i) MiKS treatments remodel berry skin structure and cuticular wax composition

Increasing summer temperatures and recurrent drought events in the DDR represent a major challenge for maintaining grape and wine quality. In this context, the present study investigated the combined foliar application of Kl and Si in a single spray formulation, providing new insights into their complementary effects on berry structure, metabolism and wine quality under Mediterranean field conditions.

The grape berry skin (the exocarp) consists of epidermal tissues covered by a cuticle, whose protective properties are largely determined by its cuticular wax composition. As well as regulating water loss and cuticular transpiration, the skin contributes to the berry’s mechanical stability, hydrophobicity, photoprotection, and defense against pathogens such as *Botrytis cinerea* (Dimopoulos et al., 2020; Zhang et al., 2021). It also constitutes the main reservoir of phenolic compounds, pigments and aroma precursors (Gambetta et al., 2021; Domanda et al., 2024).

In the present study, foliar applications combining Kl and Si induced significant anatomical changes in skin of Touriga Nacional berries, particularly through increased cuticle thickness at both veraison and harvest. The enhancement observed across MiKS treatments, especially under MiKS 4, suggests that cuticle deposition responds to MiKS application and may reinforce the berry’s external protective barrier (Ollat et al., 2002; Chang and Keller, 2021). Under Mediterranean climatic conditions, characterized by high temperatures, intense solar radiation and limited water availability during ripening, thicker cuticles may represent an important adaptive trait to reduce water loss and preserve berry integrity (Rienth et al., 2021; Rustioni et al., 2023). Previous studies have associated cuticle thickening with acclimatation to drought and heat stress, acting as a physical barrier against water loss, sunburn, and berry shriveling (Conde et al., 2024; Rogiers et al., 2022; Gambetta et al., 2021). Therefore, the cuticle reinforcement observed in this work may contribute to enhanced protection of berry tissues under summer stress conditions typical of the region.

The observed increase in cuticle thickness under MiKS treatments may reflect combined application of Kl and Si. Indeed, Kl particles films can reduce radiation load and berry temperature by increasing reflectance, thereby mitigating heat stress. The observations are consistent with previous reports describing Si mediated structural reinforcement in other plant species, although Si deposition in cell walls contributing to increased mechanical strength and reduced permeability (Dinis et al., 2018; Frioni et al., 2019; Shen et al., 2022; Wang et al., 2021). The increase in cuticle thickness under MiKS treatments is in line with previous studies showing that silicon can improve fruit firmness and skin resistance, while Kl particle films can mitigate thermal load and help maintain berry integrity under summer stress (Karagiannis et al., 2021; Wang et al., 2026).

Notably, the increase in cuticle thickness in the present study was not accompanied by an increase in the cuticular wax content, suggesting that berry cuticle thickening resulted from compositional remodeling rather than enhanced wax deposition.

This interpretation is supported by GC–MS analysis of cuticular waxes, which revealed a marked shift in wax composition between Control and MiKS treated berries. While Control was predominantly characterized by carboxylic acids, MiKS-treated berries showed a marked enrichment in pentacyclic triterpenoids, which became the predominant wax class across all MiKS treatments. Multivariate analysis further supported this pattern, separating Control from MiKS-treated berries and identifying oleanolic acid as one of the major compounds contributing to treatment differences. Interestingly, a comparable increase in cuticle thickness was also observed in leaves subjected to same MiKS formulations. A similar response has also been reported in berries of another grapevine variety (Touriga Franca) exposed to same MiKS applications, where increased cuticle thickness was likewise observed (Pereira et al., 2025).

This consistency across vegetative and reproductive tissues, as well as between grapevine varieties, suggests that MiKS formulations containing Si may induce structural adjustments in grape cuticles. Previous studies have reported that Si can accumulate beneath the cuticle and within epidermal cell walls, contributing to tissue reinforcement and altered cuticle organization in leaves (Hattori et al., 2007; Debona et al., 2017; Li et al., 2025). Although Si localization was not directly assessed in berry cuticles in the present study, the observed increase in cuticle thickness, together with the shift toward pentacyclic triterpenoids, may reflect cuticle remodeling rather than increased wax deposition. Triterpenoids, particularly oleanolic acid, are major constituents of grape berries cuticular waxes, and have been associated with modifications in cuticular architecture, permeability, and mechanical properties during berry development (Pensec et al., 2014; Misra, 2015; Trivedi et al., 2019; Dimopoulos et al., 2020; Zhang et al., 2021).

### ii) Synergetic effects of MiKS on water status and berry metabolism

The δ¹³C value of grape must is a reliable indicator of the physiological effects of foliar treatments on grapevines, as it integrates plant responses over the ripening period until harvest, reflecting the cumulative effects on stomatal conductance and discrimination against ¹³C (Gaudillère et al., 2002; Bchir et al., 2016). This isotopic discrimination results from the preferential fixation of ¹²CO_2_ over ¹³CO_2_ during carbon assimilation by Rubisco, a process regulated by stomatal conductance. Under water deficit conditions, reduced stomatal opening decreases the Ci/Ca ratio, thereby reducing discrimination against ¹³C and resulting in less negative δ¹³C values (Farquhar et al., 1989). Although limited to a single year (2024), the results indicate that grapevines treated with MiKS 2 exhibited more negative δ¹³C values than the Control, suggesting higher isotopic discrimination, and a more favorable vine water status, which may reflect improved stomatal conductance. This pattern is consistent with the previous studies showing that Kl can reduce canopy and berry heating, lower vapor pressure deficit, favoring stomatal opening and carbon assimilation, while Si has been associated with improved water use efficiency, and stress mitigation in plants (Conde et al., 2016; Zhu et al., 2019; Glenn and Puterka, 2004; Dinis et al., 2020; Brillante et al., 2020). In contrast, MiKS 4 showed less negative δ¹³C values, closer to the Control, suggesting that higher Si concentration did not further improve water stress mitigation despite promoting structural berry adjustments.

This interpretation is consistent with leaf physiological responses previously reported from same field experiment and growing season, in which grapevine treated with same MiKS formulations, particularly MiKS 2 and MiKS 3, improved leaf water potential and stomatal conductance, whereas the highest Si concentration (8%) showed physiological responses closer to the untreated vines during periods of higher evaporative demand (Monteiro et al., 2026). The main quality parameters, such as °Brix, pH, and total acidity, were not significantly affected by the foliar treatments. This suggests that MiKS applications did not significantly impact technological ripening.

The maintenance of this balance under these treatments suggests that the applied foliar strategy can be integrated as a mitigation approach to summer stress without disturbing the balance between sugars accumulation, acidity, and color, which is particularly relevant for high-quality red varieties such as Touriga Nacional in the DDR. However, the reduction in volatile acidity observed in the MiKS treated plants, particularly in 2024, may be associated with improved berry integrity. Although microcracks were not evaluated in the present study, the increased cuticle thickness observed under MiKS treatments could potentially reduce their occurrence, thereby limiting microbial entry into the berry. Since these fissures are recognized as important entry points for microorganisms, such as *Botrytis cinerea* and acetic acid bacteria, this mechanism may partly explain the lower volatile acidity observed in MiKS treated berries (Váczy et al., 2024; Nepi et al., 2025).

In turn, YAN is a key indicator of must fermentative capacity and wine quality potential, as it represents a limiting factor for yeast metabolism, particularly for *Saccharomyces cerevisiae* during alcoholic fermentation (Bouloumpasi et al., 2023). Its quantification is therefore an important enological tool for anticipating supplementation needs or adjustments in vinification conditions to avoid sluggish or stuck fermentations. In 2023, reductions in YAN were observed in MiKS 2 and MiKS 3, but no significant differences were detected in 2024. Considering that these treatments were associated with a more favorable water status and stomatal conductance, as reflected by δ¹³C and physiological measurements, the lower YAN observed in 2023 may reflect altered nitrogen partitioning during ripening, possibly associated with enhanced metabolic activity (Christofi et al., 2022). However, this response was not consistent across years, suggesting that environmental conditions strongly modulate nitrogen dynamics in the berry.

Regarding berry phenolic compounds, the absence of statistically significant differences in TCD, total phenols, and TRP indicates that MiKS application did not compromise phenolic ripening nor delay maturation. Since these compounds are directly relevant to wine quality, the maintenance of these parameters under the applied treatments represents an important agronomic and oenological result. The reduction in malic acid observed in 2023 under MiKS 2 and MiKS 3 in veraison does not appear to be associated with greater thermal or water stress. On the contrary, these plants showed better water status and more favorable δ¹³C values, reflecting greater intrinsic water use efficiency and superior stomatal regulation. Thus, at this stage, the reduction in malic acid, together with the significant changes observed in the phenylpropanoid pathway, particularly in total phenols and anthocyanins, may be associated with metabolic adjustments accompanying secondary metabolite accumulation (Sweetman et al., 2009; Volschenk et al., 2006; Zhan et al., 2023).

### iii) Oenological and aromatic implications of MiKS applications

Metabolomic analysis revealed significant changes in berry secondary metabolism, particularly metabolites derived from the phenylpropanoid pathway. MiKS 3 and MiKS 4 induced a higher accumulation of condensed flavan-3-ols and tannins, together with the reduced levels of several anthocyanin-derived metabolites compared to the Control. These metabolites were also principal contributors to treatment separation in the PCA, suggest that MiKS foliar applications may be associated with changes in the berry phenolic profile by redirecting flavonoid metabolic flux from the anthocyanin branch towards the flavan-3-ol/procyanidin branch thereby modifying the balance between different branches of the flavonoid pathway, as previously for Kl under summer stress conditions by Conde et al. (2016). The observed increase in aromatic amino acids, such as phenylalanine and tyrosine, further supports this interpretation. Phenylalanine is a primary precursor of the phenylpropanoid pathway through phenylalanine ammonia-lyase (PAL) activity, which channels carbon towards flavonoids, tannins, and other phenolic compounds (Gouot et al., 2019; Oosalo et al., 2024; Ninkuu et al., 2025). Thus, the higher levels observed under MiKS 3 and MiKS 4 may indicate an increased commitment of phenylalanine-derived metabolism towards phenolic biosynthesis. These findings are consistent with previous studies associating Kl with enhanced PAL activity and flavonoid biosynthesis under summer stress conditions (Conde et al., 2016) and with transcriptomic evidence that Si and related particle films can upregulate PAL, chalcone synthase (CHS) and other early phenylpropanoid genes, thereby increasing flux into the flavonoid branch (Afifi et al., 2023; Küskü, 2026). In contrast, concomitant several anthocyanins showed reduced levels under MiKS treatments, particularly under MiKS 4, is consistent with deferential modulation of flavonoid pathway. Anthocyanins represent the final branch of the flavonoid pathway responsible for berry color development, and their biosynthesis is strongly influenced by environmental factors such as temperature, radiation, and plant water status (de Rosas et al., 2022; Dou et al., 2024; Zhao et al., 2024). While previous studies have reported increased anthocyanin accumulation in Touriga Nacional berries following Kl application (Conde et al., 2016; Dinis et al., 2016). MiKS 3 and MiKS 4 showed the opposite trend, with higher accumulation of flavan-3-ols and procyanins accompanied by reduced levels of several anthocyanins derivatives. The reduction in several anthocyanin derivatives suggests a differential modulation of the flavonoid pathway, favoring the accumulation of flavan-3-ols and procyanins over specific anthocyanin derivatives (Dou et al., 2024; Mao et al., 2025; Murcia et al., 2025). The differences observed among MiKS treatments may be associated with the complementary effects of Kl and Si on vine physiology and berry microclimate, ultimately modulating berry metabolic response during ripening. Since Kl has been shown to mitigate heat stress and modulate phenylpropanoid metabolism, its combination with Si may have contributed to the differential accumulation of phenolic metabolites observed in the present study (Conde et al., 2016). The more pronounced metabolic changes observed under MiKS 3 and MiKS 4, compared with MiKS 2, suggest that higher Si concentrations intensified berry metabolic remodeling, although this was not consistently accompanied by improved vine water status.

In particular, the progressive increase in flavanols and procyanidins between treatments may reflect a dependent response associated with improved stress mitigation and remodeling of berry metabolism.

The VOCs analysis also provides insights into whether the physiological and metabolic responses induced by foliar treatments during berry development are reflected in the final aromatic profile of the resulting Port wine. Wine volatile composition arises from the interaction between berry-derived metabolic precursors and fermentative processes, and its characterization helps to elucidate how foliar treatments can influence wine aroma by modulating grapevine physiology during berry development (Dinis et al., 2024; Caldeira et al., 2025; Gastão-Muchecha et al., 2025; Moro et al., 2025). Consequently, the volatile profile of the resulting wines reflects both berries derived metabolic changes and yeast driven transformations occurring during alcoholic fermentation (Chávez-Márquez et al., 2025; Agnolo et al., 2026). A high qualitative similarity among treatments was observed, as most of the 76 identified compounds were common to all wines. This finding indicates that MiKS foliar treatments did not induce major qualitative shifts in wine aroma composition, but rather quantitative changes in the relative abundance of volatile families, thereby preserving the varietal identity of the Touriga Nacional variety. This aspect is particularly relevant considering that agronomic practices aimed at mitigating summer stress may alter grape metabolism and, consequently, affect the wine typicity.

Among the volatile families, wines obtained from MiKS 2 and MiKS 4 generally exhibited higher levels of several esters and alcohols, particularly compounds associated with fruity and floral sensory attributes, such as isoamyl acetate, ethyl hexanoate, and 2-phenylethyl alcohol (Boss et al., 2015; Tarko and Duda, 2024). Additionally, MiKS 4 wines were characterized by higher levels of furfural and acetic acid, indicating a somewhat distinct volatile signature compared with the remaining treatments. Esters are primarily formed during alcoholic fermentation through yeast metabolism, and their production is strongly influenced by grape composition, including the sugar availability, YAN, and metabolic precursors present in the berries (Prusova et al., 2022; Dinis et al., 2024; Gastão-Muchecha et al., 2025). Therefore, treatment related differences observed in wine VOCs likely reflected the interaction between metabolic adjustments induced by MiKS application, particularly associated with amino acids and phenylpropanoid metabolism, and yeast fermentative activity, rather than the exclusive effects of either process. Since only the final wine volatile profile was analyzed, the present study does not allow discrimination between changes resulting directly from berry metabolism and those arising during alcoholic fermentation. These findings are partially consistent with previous work by Dinis et al. (2024) in Touriga Nacional variety, where foliar application of Kl increased the abundance of wine volatile compounds (41 out of 95 detected), particularly fruity esters, alcohols, and phenolic derived compounds. By reducing temperature and radiation exposure, Kl helps preserve aroma precursors and maintain metabolic conditions favourable for aroma development during fermentation, while preserving varietal typicity (Fernandes et al., 2015; Gashu et al., 2022).

Together, these approaches would contribute to a more comprehensive understanding of the structural and physiological mechanisms underlying the protective effects of MiKS formulations under Mediterranean summer conditions.

### iv) Study limitations and future perspectives

Although the present study provides a comprehensive characterization of the effects of MiKS formulations on berry structure, cuticular wax composition, metabolite profiles, and wine volatile composition, some limitations should be acknowledged. Most of the advanced analyses, including berry skin histology, cuticular wax characterization, metabolomics, δ¹³C, and wine VOC profiling, were conducted only during the 2024 growing season. Therefore, these results should be interpreted in the context of that growing season and environmental conditions. In addition, berry temperature was not measured directly, preventing assessment of the effect of MiKS treatments on berry thermal regulation.

Future studies should investigate silicon localization within the berry skin to determine whether silicon deposition contributes to the increased cuticle thickness observed in the present study. Furthermore, evaluating whether the increased cuticle thickness induced by MiKS formulations is associated with reduced microcrack formation, lower cuticle permeability, and improved berry integrity would provide further insight into the mechanisms by which MiKS enhances berry protection under summer stress.

## 5 Conclusion

This study evaluated the effects of combined Kl and Si (MiKS) foliar applications on structural and compositional traits in Touriga Nacional berries grown under Mediterranean vineyard conditions. MiKS treatments increased cuticle thickness and altered cuticular wax composition, resulting in a higher relative abundance of pentacyclic triterpenoids, particularly oleanolic acid, without affecting the total cuticular wax content. These findings suggest that MiKS treatments were associated with structural changes in berry cuticles accompanied by modifications in wax composition. At the metabolic level, MiKS treatments were associated with changes in berry secondary metabolite profiles, including amino acid profiles, flavan-3-ols, condensed tannins, and anthocyanin-related metabolites, with magnitude of these responses varying among formulations. In addition, MiKS 2 showed more negative δ¹³C values than control, suggesting a more favorable vine water status under the conditions evaluated. Despite these structural and metabolic changes, grape quality parameters, including soluble solids, pH, and total acidity were maintained across treatments. MiKS treatments also induced quantitative changes in wine volatile composition, particularly in esters and alcohols, associated with fruity and floral aroma attributes, without markedly altering the overall volatile profile of Touriga Nacional wines.

Overall, the present study demonstrates that MiKS formulations modify berry structural and compositional characteristics while maintaining grape quality parameters under Mediterranean vineyard conditions. Among the tested formulations, MiKS 2 and MiKS 3 showed the most balanced responses across structural, metabolic, and compositional traits. Future research should further elucidate the mechanism underlying the observed structural and metabolic responses, particularly the contribution of Si to berry cuticle modifications and metabolite profile changes.

## Data Availability

The original contributions presented in the study are included in the article/**Supplementary Material**. Further inquiries can be directed to the corresponding authors.
