# Supplementary material for: Strengthening grapevine berry physiology and composition through kaolin–silicon foliar applications under summer stress in mediterranean vineyard
Source: Front Plant Sci. 2026 Aug 12;17:1906889. doi: 10.3389/fpls.2026.1906889 (PMC13508031; doi:10.3389/fpls.2026.1906889)
Supplement: Supplementary file 1 [file Supplementaryfile1.docx]

**Supplementary material**

**Table S1:** Effects of foliar application of different kaolin (Kl) and silicon (Si) formulations (MiKS): control (CTRL, water application), MiKS 2 (Kl 2% + Si 4%), MiKS 3 (Kl 2% + Si 6%), and MiKS 4 (Kl 2% + Si 8%) on the individual cuticular wax constituents of grape berries from grapevine variety Touriga Nacional during the 2024 growing season, determined by GC–MS analysis. Values are expressed as relative abundance (peak area; mean ± standard deviation; n= 5). F and p values correspond to one-way ANOVA. Different lowercase letters within the same row indicate significant differences among treatments according to Tukey’s multiple comparison test (p < 0.05).

| **Compound** | **Control** | **MiKS 2** | **MiKS 3** | **MiKS 4** | **F value** | **P value** |
| --- | --- | --- | --- | --- | --- | --- |
| Lupeol | 0.139 ± 0.005 a | 0.121 ± 0.006 ab | 0.118 ± 0.011 b | 0.134 ± 0.016 ab | 4.77 | 0.014 |
| Oleanolic acid | 1.284 ± 0.167 b | 10.4 ± 2.13 a | 10.0 ± 1.34 a | 10.6 ± 1.55 a | 46.96 | <0.001 |
| Palmitic acid | 0.417 ± 0.044 | 0.352 ± 0.039 | 0.400 ± 0.017 | 0.393 ± 0.057 | 2.18 | 0.130 |
| Tetracosanoic acid | 0.430 ± 0.078 | 0.410 ± 0.040 | 0.423 ± 0.043 | 0.403 ± 0.066 | 0.220 | 0.883 |
| Hexacosanoic acid | 0.832 ± 0.116 | 0.818 ± 0.073 | 0.803 ± 0.105 | 0.781 ± 0.138 | 0.200 | 0. 898 |
| Octacosanoic acid | 1.37 ± 0.173 | 1.23 ± 0.160 | 1.26 ± 0.180 | 1.36 ± 0.216 | 0.780 | 0.522 |
| Triacontanoic acid | 0.953 ± 0.168 | 0.888 ± 0.172 | 0.861 ± 0.123 | 0.908 ± 0.178 | 0.290 | 0.832 |
| Dotriacontanoic acid | 0.659 ± 0.124 | 0.582 ± 0.055 | 0.577 ± 0.085 | 0.540 ± 0.111 | 1.33 | 0.299 |
| Hexacosan-1-ol | 0.135 ± 0.011 | 0.124 ± 0.010 | 0.13 ± 0.028 | 0.134 ± 0.016 | 0.360 | 0.784 |
| Tetracosan-1-ol | 0.106 ± 0.033 | 0.115 ± 0.014 | 0.118 ± 0.011 | 0.124 ± 0.016 | 0.660 | 0.587 |
| Octacosan-1-ol | 0.135 ± 0.011 | 0.124 ± 0.010 | 0.13 ± 0.028 | 0.134 ± 0.016 | 0.360 | 0.784 |
| Triacontan-1-ol | 0.106 ± 0.033 | 0.115 ± 0.014 | 0.121 ± 0.008 | 0.124 ± 0.016 | 0.770 | 0.527 |
| Hexacosane | 0.067 ± 0.005 | 0.058 ± 0.007 | 0.059 ± 0.005 | 0.062 ± 0.008 | 2.20 | 0.128 |
| Heptacosane | 0.067 ± 0.005 | 0.058 ± 0.007 | 0.059 ± 0.005 | 0.062 ± 0.008 | 2.20 | 0.212 |
| Hexacosanal | 0.066 ± 0.004 | 0.058 ± 0.007 | 0.059 ± 0.005 | 0.062 ± 0.008 | 1.67 | 0.402 |
| Octacosanal | 0.066 ± 0.004 | 0.058 ± 0.007 | 0.059 ± 0.005 | 0.062 ± 0.008 | 1.67 | 0.247 |
| Oleanolic aldehyde | 0.468 ± 0.048 | 0.402 ± 0.072 | 0.411 ± 0.045 | 0.448 ± 0.096 | 1.04 | 0.402 |

**Table S2:** Berries metabolomic profile subjected to different foliar treatments (Control, MiKS2, MiKS3, and MiKS4), determined by UHPLC-MS analysis. Metabolites were grouped according to their chemical class and expressed as relative abundance values, corresponding UHPLC-MS peak areas(mean ± standard deviation; n=5). Different lowercase letters within the same row indicate significant differences among treatments according to Tukey’s multiple comparison test (*p* < 0.05).

| **Chemical**  **Class** | **Major ions**  **(m/z)** | **Metabolite** | **ID** | **Foliar treatment** | | | | ***p*_value** |
| --- | --- | --- | --- | --- | --- | --- | --- | --- |
|  |  |  |  | **Control** | **MiKS 2** | **MiKS 3** | **MiKS 4** |  |
| **Amino acids** | 116 | L-proline | M1 | 3.05 ± 0.051 c | 3.11 ± 0.063 b | 3.17 ± 0.044 a | 3.13 ± 0.082 b | 0.016 |
|  | 132 | L-leucine | M2 | 3.06 ± 0.097 b | 2.93 ± 0.135 c | 3.02 ± 0.048 b | 3.12 ± 0.050 a | 0.012 |
|  | 132 | L-isoleucine | M3 | 3.12 ± 0.174 | 3.08 ± 0.066 | 3.23 ± 0.015 | 3.19 ± 0.095 | 0.088 |
|  | 166 | L-phenylalanine | M4 | 2.75 ± 0.094 b | 2.72 ± 0.039 b | 2.95 ± 0.053 a | 3.04 ± 0.087 a | <0.001 |
|  | 182 | L-tyrosine | M5 | 2.70 ± 0.156 c | 2.74 ± 0.111 c | 2.96 ± 0.089 a | 2.86 ± 0.069 b | 0.003 |
|  | 205 | L-tryptophan | M6 | 2.89 ± 0.072 b | 2.74 ± 0.147 c | 2.84 ± 0.085 c | 3.01 ± 0.061 a | 0.001 |
| **Anthocyanins** | 449 | Cyanidin-3-*O*-galactoside | M7 | 1.86 ± 0.110 a | 1.71 ± 0.101 b | 1.83 ± 0.113 a | 1.59 ± 0.188 c | 0.008 |
|  | 463 | Peonidin-3-*O*-glucoside | M8 | 2.75 ± 0.097 | 2.69 ± 0.109 | 2.76 ± 0.076 | 2.64 ± 0.159 | 0.262 |
|  | 465 | Delphinidin-3-*O*-glucoside | M9 | 2.67 ± 0.090 a | 2.48 ± 0.025 a | 2.66 ± 0.144 a | 2.51 ± 0.222 a | 0.049 |
|  | 479 | Petunidin-3-*O*-glucoside | M10 | 2.85 ± 0.105 a | 2.67 ± 0.028 c | 2.84 ± 0.113 a | 2.77 ± 0.126 b | 0.017 |
|  | 493 | Malvidin-3-*O*-glucoside | M11 | 3.79 ± 0.098 | 3.72 ± 0.121 | 3.74 ± 0.126 | 3.65 ± 0.097 | 0.189 |
|  | 535 | Malvidin-3-*O*-(6-*O-*acetyl)-glucoside | M12 | 3.69 ± 0.071 a | 3.53 ± 0.124 b | 3.58 ± 0.088 b | 3.51 ± 0.108 c | 0.025 |
|  | 625 | Petunidin-3-*O*-(6-p-coumaroyl)-glucoside | M13 | 2.82 ± 0.076 a | 2.65 ± 0.117 c | 2.78 ± 0.050 b | 2.47 ± 0.130 d | <0.001 |
|  | 639 | Malvidin-3-*O*-(6-p-coumaroyl)-glucoside | M14 | 3.84 ± 0.083 a | 3.74 ± 0.089 b | 3.68 ± 0.123 c | 3.58 ± 0.070 d | <0.001 |
|  | 655 | Malvidin-3,5-*O*-diglucoside | M15 | 1.52 ± 0.094 a | 1.44 ± 0.038 b | 1.48 ± 0.070 a | 1.33 ± 0.091 c | 0.002 |
|  | 491 | Cyanidin-3-*O*-(6-*O*-acetyl)-glucoside | M35 | 1.16 ± 0.040 b | 1.19 ± 0.100 b | 1.49 ± 0.142 a | 1.16 ± 0.251 b | 0.003 |
|  | 521 | Petunidin-3-*O*-(6-*O*-acetyl)-glucoside | M36 | 2.36 ± 0.156 b | 2.17 ± 0.138 d | 2.42 ± 0.065 a | 2.23 ± 0.083 c | 0.004 |
| **Phenolic acids** | 169 | Gallic acid | M16 | 0.580 ± 0.135 b | 0.444 ± 0.055 c | 0.695 ± 0.140 a | 0.725 ± 0.088 a | 0.001 |
|  | 295 | Coutaric acid | M20 | 2.69 ± 0.052 | 2.69 ± 0.054 | 2.68 ± 0.133 | 2.64 ± 0.104 | 0.744 |
|  | 311 | Caftaric acid | M21 | 2.84 ± 0.054 | 2.84 ± 0.055 | 2.75 ± 0.144 | 2.78 ± 0.049 | 0.171 |
|  | 325 | Fertaric acid | M22 | 1.66 ± 0.057 | 1.59 ± 0.114 | 1.69 ± 0.040 | 1.62 ± 0.074 | 0.149 |
| **Flavan-3-ols** | 289 | Catechin | M18 | 1.68 ± 0.112 b | 1.61 ± 0.055 c | 1.78 ± 0.119 a | 1.79 ± 0.095 a | 0.013 |
|  | 289 | Epicatechin | M19 | 1.41 ± 0.211 | 1.49 ± 0.100 | 1.58 ± 0.200 | 1.60 ± 0.144 | 0.236 |
|  | 441 | Catechin gallate | M23 | 0.719 ± 0.127 b | 0.706 ± 0.128 b | 1.04 ± 0.056 a | 0.958 ± 0.149 a | <0.001 |
| **Procyanidins (condensed tannins)** | 577 | Procyanidin B1 | M28 | 1.78 ± 0.095 | 1.77 ± 0.019 | 1.76 ± 0.155 | 1.89 ± 0.189 | 0.292 |
|  | 577 | Procyanidin B2 | M29 | 1.10 ± 0.077 c | 1.25 ± 0.142 b | 1.42 ± 0.104 a | 1.35 ± 0.155 a | 0.001 |
|  | 577 | Procyanidin B3 | M30 | 0.994 ± 0.027 | 0.959 ± 0.014 | 1.06 ± 0.148 | 1.06 ± 0.135 | 0.219 |
|  | 577 | Procyanidin B4 | M31 | 0.892 ± 0.034 c | 0.985 ± 0.118 b | 1.12 ± 0.109 a | 1.14 ± 0.119 a | 0.001 |
|  | 865 | Procyanidin trimer 1 | M33 | 1.07 ± 0.094 b | 0.973 ± 0.014 b | 1.04 ± 0.080 b | 1.28 ± 0.064 a | <0.001 |
|  | 865 | Procyanidin trimer 2 | M34 | 1.04 ± 0.536 | 0.829 ± 0.122 | 0.795 ± 0.095 | 0.880 ± 0.096 | 0.479 |
|  | 729 | Procyanidin gallate | M38 | 0.968 ± 0.550 | 0.502 ± 0.043 | 0.793 ± 0.126 | 0.696 ± 0.148 | 0.077 |
| **Flavonols** | 432 | Kaempferol glucoside | M24 | 1.61 ± 0.038 | 1.40 ± 0.304 | 1.41 ± 0.139 | 1.60 ± 0.204 | 0.140 |
|  | 463 | Quercetin-3-*O*-glucoside | M25 | 2.09 ± 0.121 a | 1.98 ± 0.207 b | 1.85 ± 0.082 c | 2.11 ± 0.141 a | 0.020 |
|  | 477 | Quercetin-3-*O*-glucuronide | M26 | 1.81 ± 0.260 | 1.74 ± 0.114 | 2.03 ± 0.253 | 1.97 ± 0.412 | 0.270 |
|  | 479 | Myrcetin hexoside | M27 | 2.49 ± 0.016 | 2.45 ± 0.080 | 2.47 ± 0.077 | 2.40 ± 0.087 | 0.191 |
|  | 539 | Kaempferol-3-*O*-rutinoside | M32 | 1.21 ± 0.065 b | 1.07 ± 0.101 c | 1.26 ± 0.052 a | 0.901 ± 0.136 d | <0.001 |
| **Organic acids** | 191 | Citric acid | M17 | 2.66 ± 0.112 b | 2.50 ± 0.039 d | 2.71 ± 0.057 a | 2.55 ± 0.044 c | <0.001 |
| **Stilbenes** | 398 | Piceid | M37 | 0.586 ± 0.172 | 0.676 ± 0.120 | 0.676 ± 0.135 | 0.673 ± 0.117 | 0.611 |

**Table S3.** Volatile organic profile of wines subjected to different foliar treatments (Control, MiKS2, MiKS3, and MiKS4), determined by HS-SPME-GC-MS. Identified compounds were classified according to their chemical class and characterised based on retention time (RT), retention index (RI), reported RI, major ions (m/z), aroma descriptors, and identification methods.

| **Chemical**  **class** | **Compounds** | **CAS** | **RT** | **RI** | **Reported RI** | **Major ions**  **(m/z)** | **Aroma**  **descriptors** | **Identification** |
| --- | --- | --- | --- | --- | --- | --- | --- | --- |
|  |  |  |  |  |  |  |  |  |
| **Alcohols** | 1-Propanol | 71-23-8 | 3.82 | 1028 | 1038 | 59/60/61 | Fruit, Plastic, Pungent, Musty | MainLib |
|  | Isobutanol | 78-83-1 | 5.19 | 1096 | 1108 | 55/57/59/73/74 | Solvent, glue, alcohol, licorice | MainLib |
|  | Isoamyl alcohol | 123-51-3 | 9.09 | 1206 | 1206 | 55/57/69/70/71 | Pungent, balsamic, malty, burnt | MainLib |
|  | 1-Pentanol | 71-41-0 | 9.93 | 1222 | 1249 | 55/70/88/99 | Green, wax | STD |
|  | 1-Hexanol | 111-27-3 | 16.7 | 1342 | 1353 | 55/56/69 | Herbaceous, grass, woody | STD |
|  | *cis*-3-Hexen-1-ol | 928-96-1 | 18.4 | 1368 | 1379 | 55/67/82/98 | Green, bitter, fatty | STD |
|  | 2-Ethylhexan-1-ol | 104-76-7 | 26.1 | 1479 | 1492 | 57/70/83 | Citrus, fresh, floral, sweet | MainLib |
|  | 1-Octanol | 111-87-5 | 30.9 | 1546 | 1566 | 56/69/70/84 | Metallic, sulfury, toasted, herbal | MainLib |
|  | 2,3-Butanediol | 513-85-9 | 32.1 | 1562 | 1582 | 57/75/90 | Fruity | MainLib |
|  | 2-Methylhexadecan-1-ol | 2490-48-4 | 37.2 | 1635 | - | 55/57/70/83/97/111 | - | MainLib |
|  | 1-Nonanol | 143-08-8 | 38.2 | 1648 | 1619 | 56/70/83/97 | Fat, green, fruity | MainLib |
|  | 2-Dodecanol | 10203-28-8 | 48.1 | 1813 | - | 55/69/83/97/111 | - | - |
|  | Benzyl alcohol | 100-51-6 | 49.2 | 1828 | 1855 | 56/71/77/79/108 | Fruity, walnut, bitter almond | STD |
|  | 2-Phenylethanol | 60-12-8 | 50.6 | 1874 | 1931 | 65/91/92/122 | Honey, sweet, yeast, floral, spicy | MainLib |
| **Esters** | Ethyl acetate | 141-78-6 | 2.03 | 884(*) | 901 | 61/70/73/88 | Pineapple, fruity, solvent, balsamic | STD |
|  | Ethyl isobutyrate | 97-62-1 | 2.78 | 960 | 965 | 71/73//88/89/116 | Fruit, Strawberry | STD |
|  | Isobutyl acetate | 110-19-0 | 3.17 | 993 | 1005 | 55/56/71/73 | Apple, Banana | STD |
|  | Ethyl butanoate | 105-54-4 | 3.66 | 1020 | 1053 | 70/71/73/88/89/101 | Apple, Banana | STD |
|  | Ethyl 2-methylbutanoate | 7452-79-1 | 3.96 | 1035 | 1061 | 57/74/85/102/115 | Fruity, green apple | STD |
|  | *Ethyl isovalerate* | 108-64-5 | 4.25 | 1049 | 1070 | 57/60/70/85/88 | Cashew, fruit, anise, blackcurrant | MainLib |
|  | Isoamyl acetate | 123-92-2 | 5.46 | 1105 | 1123 | 55/61/70/73/87 | Banana, fruity, sweet | STD |
|  | Ethyl pentanoate | 539-82-2 | 5.82 | 1115 | 1147 | 57/69/73/85/88/101 | Fruit, orange, grass, mint | MainLib |
|  | Amyl acetate | 628-63-7 | 6.52 | 1135 | 1175 | 61/70/73/87/91 | Herbaceous | MainLib |
|  | Ethyl hexanoate | 123-66-0 | 9.70 | 1217 | 1227 | 60/73/80/99/101 | Fruit, green apple, banana, brandy | STD |
|  | Hexyl acetate | 142-92-7 | 11.6 | 1254 | 1265 | 56/61/69/84 | Apple, cherry, pear, floral | STD |
|  | Ethyl 3-hexenoate | 2396-83-0 | 13.5 | 1290 | 1269 | 55/69/77/88/142 | Fruity, green, sweet, pineapple | MainLib |
|  | Ethyl heptanoate | 106-30-9 | 15.0 | 1316 | 1332 | 55/70/88/101/113 | Fruity | MainLib |
|  | Ethyl lactate | 97-64-3 | 15.6 | 1325 | 1353 | 56/75/103 | Ethereal, buttery | MainLib |
|  | Methyl octanoate | 111-11-5 | 18.6 | 1371 | 1378 | 57/67/87/115/127 | Fruity, green | MainLib |
|  | Ethyl 2-methyloctanoate | 30982-02-6 | 21.2 | 1410 | 1388 | 57/74/88/102/115/129 | - | MainLib |
|  | Ethyl octanoate | 106-32-1 | 21.9 | 1420 | 1427 | 57/60/70/88/101/127 | Banana, pear, brandy | STD |
|  | Pentyl hexanoate | 540-07-8 | 27.0 | 1492 | 1501 | 70/85/99/117 | Sweet, green, fruit | MainLib |
|  | Propyl octanoate | 624-13-5 | 27.7 | 1501 | 1530 | 55//61/101/127/145 | Winey, oily, brandy | MainLib |
|  | Vinyl caprylate | 818-44-0 | 28.1 | 1506 | - | 57/109/127 | - | MainLib |
|  | Ethyl nonanoate | 123-29-5 | 29.0 | 1518 | 1528 | 55/60/70/88/101/141 | Fruity, rose, waxy, tropical | MainLib |
|  | Butyrolactone | 96-48-0 | 32.4 | 1567 | 1640 | 56/85/86 | Sweet, aromatic, caramel | MainLib |
|  | Ethyl decanoate | 110-38-3 | 36.3 | 1621 | 1628 | 55/60/70/88/101 | Brandy, fruity, grape | STD |
|  | 3-Methylbutyl octanoate | 2035-99-6 | 37.5 | 1638 | 1658 | 55/70/127 | Sweet, fruity, waxy, pineapple | MainLib |
|  | Diethyl butanedioate | 123-25-1 | 38.6 | 1654 | 1690 | 55/73/101/129 | Fabric, fruit, watermelon, flower, potato | MainLib |
|  | Octyl ether | 629-82-3 | 44.3 | 1742 | 1761 | 57/71/83/111 | - | MainLib |
|  | Phenethyl acetate | 103-45-7 | 46.3 | 1778 | 1687 | 91/104 | Rose, honey | STD |
|  | Ethyl laurate | 106-33-2 | 48.7 | 1826 | 1822 | 55/70/88/101 | Mango | MainLib |
|  | Isopropyl myristate | 110-27-0 | 55.8 | 2020 | 2041 | 57/60/73/102/228 | Faint, oily, fatty | MainLib |
|  | Ethyl tetradecanoate | 124-06-1 | 56.0 | 2028* | 2029 | 55/88/101/157 | Oil, ethereal, violet | MainLib |
|  | Ethyl palmitate | 628-97-7 | 61.8 | 2197* | 2229 | 55/73/88/101 | Waxy, mild, sweet | MainLib |
| **Terpenes** | Linalool | 78-70-6 | 30.2 | 1636 | 1551 | 55/71/93/121 | Citrus, floral | STD |
|  | α-Terpineol | 98-55-5 | 39.7 | 1670 | 1711 | 59/67/81/93/121/136 | Lilac, floral, sweet | STD |
| **Furans** | Furfural | 98-01-1 | 22.7 | 1432 | 1445 | 95/96 | Burn almond | STD |
| **Aldehydes** | Benzaldehyde | 100-52-7 | 25.9 | 1476 | 1489 | 51/77/105 | Bitter almond, smoky | STD |
| **Ketones** | 2,3-Pentanedione | 600-14-6 | 4.07 | 1040 | 792 | 57/100 | Caramel, sweet, buttery | MainLib |
|  | 2-Octanone | 111-13-7 | 11.8 | 1259 | 1276 | 58/85/113/128 | Herbal | STD |
|  | 6-Methyl-5-hepten-2-one | 110-93-0 | 14.9 | 1323 | 1365 | 55/69/75/87/103 | Mushroom, earthy, vinyl, boiled fruit | MainLib |
|  | 2-Dodecanone | 6175-49-1 | 40.1 | 1676 | 1698 | 58/71/85 | Fruity, citrus, floral, orange | MainLib |
| **Acids** | Acetic acid | 64-19-7 | 22.5 | 1428 | 1442 | 60/61 | Vinegar, sour, pungent | STD |
|  | Caprylic acid | 124-07-2 | 56.8 | 2052* | 2098 | 55/60/73/101 | Fatty, cheesy, fresh, moss | MainLib |
| **Unknown compounds** | Unknown 1 | - | 6.61 | 1138 | - | 55/56/57/70 | - | - |
|  | Unknown 2 | - | 6.93 | 1147 | - | 55/61/70/73/87 | - | - |
|  | Unknown 3 | - | 7.07 | 1151 | - | 57/70/91 | - | - |
|  | Unknown 4 | - | 7.64 | 1167 | - | 55/68/74/93/107 | - | - |
|  | Unknown 5 | - | 7.96 | 1176 | - | 55/69/70/73/87 | - | - |
|  | Unknown 6 | - | 8.63 | 1195 | - | 55/56/57/69/83/105/114 | - | - |
|  | Unknown 7 | - | 11.2 | 1247 | - | 55/60/70/88/99/105 | - | - |
|  | Unknown 8 | - | 11.8 | 1257 | - | 55/61/73/88/99 | - | - |
|  | Unknown 9 | - | 12.7 | 1274 | - | 55/77/105 | - | - |
|  | Unknown 10 | - | 14.5 | 1308 | - | 55/69/71/80/93 | - | - |
|  | Unknown 11 | - | 15.4 | 1322 | - | 55/69/73/88/97 | - | - |
|  | Unknown 12 | - | 17.2 | 1350 | - | 56/67//82/97 | - | - |
|  | Unknown 13 | - | 17.6 | 1355 | - | 55/56/79/83/98 | - | - |
|  | Unknown 14 | - | 23.5 | 1443 | - | 57/72/99/117 | - | - |
|  | Unknown 15 | - | 26.3 | 1482 | - | 60/70/88/101 | - | - |
|  | Unknown 16 | - | 27.2 | 1494 | - | 57/69/99/107/121 | - | - |
|  | Unknown 17 | - | 47.0 | 1789 | - | 57/71/83/112/133 | - | - |
|  | Unknown 18 | - | 48.9 | 1832 | - | 57/69/83//88/101 | - | - |
|  | Unknown 19 | - | 49.4 | 1840 | - | 60/70/73/87/97 | - | - |
|  | Unknown 20 | - | 61.5 | 2188* | - | 55/73/83/97/102/239 | - | - |
|  | Unknown 21 | - | 64.0 | 2262* | - | 73/105/149/177 | - | - |
|  | RT, retention time; RI, retention index; STD, identification by analytical standard; MainLib, identification based on mass spectral library. | | | | | | | |
